# Differential Effects of the G-Protein-Coupled Estrogen Receptor (GPER) on Rat Embryonic (E18) Hippocampal and Cortical Neurons

**DOI:** 10.1523/ENEURO.0475-21.2022

**Published:** 2022-07-14

**Authors:** Kyle Pemberton, Martina Rosato, Cass Dedert, Chelsea DeLeon, Christopher Arnatt, Fenglian Xu

**Affiliations:** 1 The Department of Biology, College of Arts and Sciences, Saint Louis University, St. Louis, MO 63103; 2 The Henry and Amelia Nasrallah Center for Neuroscience, Saint Louis University, St. Louis, MO 63104; 3 The Department of Pharmacology and Physiology, School of Medicine, Saint Louis University, St. Louis, MO 63104; 4 The Department of Chemistry, College of Arts and Sciences, Saint Louis University, St. Louis, MO 63103

**Keywords:** electrophysiology, estrogen, G-protein-coupled estrogen receptor (GPER/GPR30), hippocampus, neurodevelopment, transcriptome

## Abstract

Estrogen plays fundamental roles in nervous system development and function. Traditional studies examining the effect of estrogen in the brain have focused on the nuclear estrogen receptors (ERs), ERα and ERβ. Studies related to the extranuclear, membrane-bound G-protein-coupled ER (GPER/GPR30) have revealed a neuroprotective role for GPER in mature neurons. In this study, we investigated the differential effects of GPER activation in primary rat embryonic day 18 (E18) hippocampal and cortical neurons. Microscopy imaging, multielectrode array (MEA), and Ca^2+^ imaging experiments revealed that GPER activation with selective agonist, G-1, and nonselective agonist, 17β-estradiol (E2), increased neural growth, neural firing activity, and intracellular Ca^2+^ more profoundly in hippocampal neurons than in cortical neurons. The GPER-mediated Ca^2+^ rise in hippocampal neurons involves internal Ca^2+^ store release via activation of phospholipase C (PLC) and extracellular entry via Ca^2+^ channels. Immunocytochemistry results revealed no observable difference in GPER expression/localization in neurons, yet real-time qPCR (RT-qPCR) and Western blotting showed a higher GPER expression in the cortex than hippocampus, implying that GPER expression level may not fully account for its robust physiological effects in hippocampal neurons. We used RNA sequencing data to identify distinctly enriched pathways and significantly expressed genes in response to G-1 or E2 in cultured rat E18 hippocampal and cortical neurons. In summary, the identification of differential effects of GPER activation on hippocampal and cortical neurons in the brain and the determination of key genes and molecular pathways are instrumental toward an understanding of estrogen’s action in early neuronal development.

## Significance Statement

Studies of estrogen function via a non-nuclear G-protein-coupled estrogen receptor (GPER/GPR30) in the brain have primarily focused on mature neurons and neuroprotective actions with little investigation into the role of GPER in early neural development. In this work, we discover differential effects of GPER on early neurite outgrowth, neuronal activity, and intracellular calcium (Ca^2+^) signaling in primarily cultured rat embryonic [embryonic day (E)18] hippocampal and cortical neurons. This study further highlights distinct, transcriptomic genes and pathways that are regulated by GPER agonists in early developing hippocampal and cortical neurons. These results advance our fundamental understanding of estrogen functions via GPER signaling in different (hippocampal vs cortical) neurons during early neuronal development. This knowledge is also instrumental for therapeutics for GPER-related neurodevelopmental disorders.

## Introduction

The steroid hormone estrogen plays crucial roles in the nervous system, ranging from developmental function to neuroprotection after injury (Miranda et al., 1994; Kajta and Beyer, 2003). The observed physiological actions of 17β-estradiol (E2), the most active form of estrogen, were originally attributed to the classical, nuclear estrogen receptors (ERs) ERα (P. Walter et al., 1985; Green et al., 1986) and ERβ (Kuiper et al., 1996). Since then, cloning of the orphan G-protein-coupled receptor (GPCR), *GPR30/GPER-1* (Carmeci et al., 1997; Kvingedal and Smeland, 1997), and subsequent deorphanization with estrogen have led to the identification of the G-protein-coupled ER (GPER; Filardo et al., 2002; Revankar et al., 2005). GPER is ubiquitously expressed in the rat brain (Brailoiu et al., 2007; Hazell et al., 2009) with a higher level of expression in the hippocampus, cortex, and hypothalamus compared with other brain regions (Hazell et al., 2009). Additionally, recent transcriptomics of adult rats has shown that the GPER transcript predominates in the brain compared with other tissues and exhibits a higher level of expression compared with the classical ERs (Hutson et al., 2019). These findings further suggest that a physiological role for GPER in the brain exists.

Despite knowledge of the importance of estrogen on the developing nervous system, direct evidence related to the role of GPER in early neuronal development is limited. Currently, research has focused on the role of GPER in mature neuronal function, primarily related to estrogen’s protective role in diseases such as Parkinson’s disease (Bourque et al., 2015; Côté et al., 2015) and ischemic stroke (Murata et al., 2013; Broughton et al., 2014). In addition, emerging research suggests that GPER may contribute to the etiology of neurodevelopmental and neuropsychiatric disorders, including autism spectrum disorder (Altun et al., 2017), schizophrenia (Gogos et al., 2015), attention deficit hyperactivity disorder (Sahin et al., 2018), anxiety (Li et al., 2013; Tian et al., 2013), and depression (McAllister et al., 2012, 2014). Moreover, the identification of *GPER-1* polymorphisms and evidence that variants of GPER may result in miscarriage during pregnancy (Tang et al., 2017) suggest that GPER plays an important role in fetal development. Further research performed in zebrafish embryos has shown a high expression of GPER in the nervous system during development (Shi et al., 2013), although the activity and mechanism have yet to be established.

The role of estrogen may not be stagnant and critical periods during development may influence gene expression. Estrogen and estrogen precursor levels in the hippocampus and cortex of rats decrease after E19 and continue to attenuate postnatally (Konkle and McCarthy, 2011), suggesting that estrogens play an important role in early neuronal development. In addition, estrogen can be produced locally in multiple brain regions in rats, including the hippocampus and cortex, and functions as a bona fide neurotrophic and neuromodulatory factor that increases synaptic plasticity within minutes to hours (Srivastava et al., 2008). Research on the pharmacology and signaling associated with GPER modulation within the brain is not well understood and may depend on the cell population (Beyer et al., 2002). For example, in adult rat cortex GPER activation induces calcium (Ca^2+^) signaling in astrocytes but not neurons (Roque and Baltazar, 2019; Roque et al., 2019). Despite these findings, few studies have focused on the effect of GPER in rat brains during early developmental stages, such as Embryonic day 18 (E18) neurons. To help fill this gap, we sought to uncover the effects of GPER during early neuronal development in rat E18 neurons originating from the hippocampus and cortex. Differential effects of targeting GPER in the hippocampus and cortex were observed with E2 and the GPER-specific agonist, G-1. The hallmark observation measured for neuronal development in our study was neurite outgrowth. We found that GPER promotes neurite outgrowth in hippocampal but not cortical neurons. Our results further revealed different physiological and signaling events between hippocampal and cortical neurons. In particular, hippocampal neurons showed greater action potential firing (neuronal activity) and intracellular Ca^2+^ oscillations than cortical neurons in response to GPER activation. These differences in physiological and signaling effects may not be attributed to the level of GPER expression. Instead, these differences may depend on more profound regulation of specific genes and signaling pathways in hippocampal cultures. RNA sequencing was used to interrogate changes in gene regulation in response to GPER activation and identify specific signaling pathways. These results are crucial in understanding the targetability of GPER during early neuronal development.

## Materials and Methods

### Animals and neuronal cell culture

Animal maintenance and experiments were conducted using protocols that follow the guidelines outlined by the National Institute of Health Animal Use Guidelines and were performed in accordance with Saint Louis University Animal Care and Use Committee. All experiments were conducted on primary neurons derived from the brains of E18 rat pups. The sex of the embryos was not determined because E18 is before the critical period (McCarthy, 2016) for masculinization, in which the testes in males begin producing large amounts of testosterone. Using neurons that have yet to be exposed to this masculinization process allows us to have confidence that sex-specific differences have not occurred and should not give confounding results based on the sex of the pups and the cells that were harvested from them.

Rat E18 primary neurons were cultured using standard dissection and culturing protocols (Pacifici and Peruzzi, 2012) in charcoal-stripped serum and phenol red-free neurobasal medium (Invitrogen, catalog #12348017) to ensure the absence of hormones or estrogenic components in medium. In short, pregnant dams were killed using CO_2_, and E18 pups were quickly removed by C-section. The brains were removed, and hippocampi and cortices were separated and placed in cold HBSS without phenol red (Invitrogen, catalog #14025092). Once all hippocampi and cortices were isolated, they were suspended separately in 50 units/ml papain solution in a 5% CO_2_, 37°C, humidified incubator for 25 min, mixing gently every 5 min. The enzyme was washed away with serum-containing medium and the tissues were mechanically triturated using flame-polished glass pipettes with progressively smaller openings. Cells were resuspended in culture medium containing neurobasal medium without phenol red, 1% L-glutamine or 1× GlutaMax (Invitrogen, catalog #35050061), 1% pen-strep, 1× B-27 supplement (Invitrogen, catalog #17504044), and 4% charcoal-stripped fetal bovine serum (FBS). Cells were then plated on glass-bottom culture dishes (MatTek, catalog #P35G-0-10-C), multichamber slides with removable wells (MatTek, catalog #CCS-8), multichamber cover glass slides (ThermoFisher, catalog #155411), or multielectrode array plates (Multichannel Systems, catalog #60MEA200/30iR-Ti) precoated with 100 μg/ml poly-D-lysine and 2 μg/ml laminin and incubated in a 5% CO_2_, 37°C, humidified incubator. To minimize glial proliferation without introducing unnecessary toxicity of an antimetabolic agent such as Ara-C, the medium was replaced with serum-free culture medium without pen-strep after 24 h in culture (HIC). The removal of pen-strep is because of findings that antibiotics can cause changes in gene expression (Ryu et al., 2017) as well as altering signaling in neurons (Bahrami and Janahmadi, 2013). The culturing surface was coated by applying a solution of 100 μg/ml poly-D-lysine and 2 μg/ml laminin in PBS to the entire culturing surface for 1 h at room temperature and then washed 3× with sterile water. Cultures were labeled with letter and number designations for blinding. Images and files were saved with the letter/number designation and not identified by treatment until analysis was completed.

### Neurite outgrowth and ImageJ neurite tracing and analysis

Comparisons between hippocampal and cortical neurons were conducted by culturing rat cortical neurons and hippocampal neurons separately at 200–300 cells/mm^2^ with 0.1% DMSO (vehicle), G-1 (100 nm), G-1 + G-15 (10 nm), E2 (100 nm), and E2 + G-15. Cultures from each group were fixed after 8, 20, 48, 72, and 96 HIC and images were taken on an Olympus IX73 inverted microscope equipped with a Retiga R1 camera (QImaging Corporation) and acquired with Micro-Manager (μManager; Edelstein et al., 2010). Concentrations of GPER agonists and antagonists are based on pharmacological profiles of agonism and antagonism according to well-established literature. Specifically, compound G-15 shows no appreciable binding to ERα or ERβ at concentrations below 10 μm (Dennis et al., 2011). Similarly, competition binding assays revealed no appreciable binding of G-1 to ERα and ERβ at concentrations up to 1 μm (Bologa et al., 2006). Therefore, we chose to use G-1 at 1–100 nm and G-15 at 10 nm; both concentrations are determined to be specific to GPER binding only.

Neurite measurements were obtained using the ImageJ package Fiji (Schindelin et al., 2012) as previously described (Imaninezhad et al., 2018; Pemberton et al., 2018; Mersman et al., 2020). In short, images were adjusted by subtracting the background to allow the processes to be more easily visualized by using the Subtract Background tool. Once backgrounds were subtracted, neurite growths were traced and labeled by cellular origin using the NeuronJ plugin (Meijering et al., 2004). Any outgrowth that was <5 μm was removed. The remaining outgrowths were labeled by their origin and summed to generate the total outgrowth per cell in R Studio (Rstudio, 2015). Neurite lengths are represented by the average total length of processes per cell as previously described (Beyer et al., 2002).

### Multielectrode array (MEA) recording of neuronal activity

Neurons were cultured on MEAs (containing electrodes in an 8 × 8 pattern) at a density of 400–500 cells/mm^2^ for a period of 14–18 d for acceptable numbers of synaptic contacts to occur (Basarsky et al., 1994; Dehorter et al., 2012). Measurements were taken for 5–10 min per sample at 20 kHz using the MEA2100-Lite headstage (Multi Channel Systems) connected to an MCS-IFB interface board. The temperature was kept at 37°C using a TC 01 temperature controller. Multi Channel Experimenter software was used to record measurements and activity spikes were identified using Multi Channel Analyzer, then exported into CSV format using Multi Channel Data Manager for further analysis. The activity was determined in R by finding the number of spikes per minute recorded by the electrode. To account for MEAs measuring activity in an area that may have a more or less dense population of cells than other electrode populations, each electrode’s activity was normalized to the activity of the electrode before treatment. Activity between treatments was compared by assessing the average amount of spikes in electrical activity per minute. The reported n numbers for these experiments represents the total number of active electrodes in each treatment group from at least three culturing experiments. Cells used for each culture were dissociated from hippocampi and cortices collected from the brains of three to six animals in each experiment.

### Ca^2+^ imaging

Primary cells cultured for 11–15 d at a density of 400–500 cells/mm^2^ were incubated in 5 μm fura-2 AM (ThermoFisher Scientific catalog #F1201) Ca^2+^ dye in HBSS without phenol red (Invitrogen, catalog #14025092) for 1 h in a 37°C incubator. Cells were then washed four times (10 min per wash) in HBSS at room temperature. Once washed, cells sat for 15–30 min on the imaging rig (Olympus IX73 inverted microscope) to allow the temperature to equilibrate and for full de-esterification of the fura-2 AM. Samples using any antagonists or pharmacological blockers (e.g., G-15, U73122, etc.) had the antagonists/blockers added after the final wash of fura-2 AM and incubated for 30 min (i.e., pretreated for ∼30 min) before being mounted on the imaging rig. The basal Ca^2+^ level in vehicle or antagonists/blockers alone was first recorded for ∼5 min before adding agonists in the continued presence of antagonists/blockers. Fura-2 was excited sequentially at 340- and 380-nm wavelengths delivered from λ XL equipped with a high-speed wavelength switcher (Sutter Instrument) via an Olympus 40× objective. The emitted fluorescence signal was collected at 510 nm by a Retiga R1 camera (QImaging). The ratio of fluorescence signal at 340 and 380 nm was obtained using the MetaFluor Imaging software (Molecular Devices) at 0.5–1 Hz and analyzed using ImageJ. At the end of each experiment, 40 mm KCl, a standard neuron stimulator, was applied to the cells. Cells responding to KCl with Ca^2+^ rise were confirmed to be neurons and are included for analysis. Failing to respond to KCl indicates the cells are either not healthy or not neurons and were not included in the analysis.

The images obtained were used to create a region of interest (ROI) around each neuron based on cell morphology. A background ROI was made separately in an empty region of the images. An ImageJ macro was developed to import each channel as an image stack to subtract the background for each image, get the average pixel intensity of each ROI, and save these values in a separate file for each channel. Results of the ImageJ macro were then imported into R to analyze the ratio between the two channels. First, the two channels’ results were imported into R and normalized. Where Ca^2+^ peaks were analyzed, peaks were determined using a modified version of the NeuronActivityTool’s (Prada et al., 2018) peak identification code. The δ (Δ) levels of rise in Ca^2+^ by agonists were shown in figures, which measured the difference in mean peak amplitudes of Ca^2+^ (340/380 ratio) in the presence of agonists + antagonists/blockers as compared with the initial basal Ca^2+^ level in the presence of antagonists/blockers alone.

### Immunocytochemistry

The expression and localization of GPER in hippocampal and cortical neurons were monitored using immunofluorescent staining and confocal microscopy techniques. Specifically, rat hippocampal and cortical neurons were cultured separately at 200–300 cells/mm^2^ for 72 HIC. Neurons were then fixed with 4% paraformaldehyde (PFA) for 20 min and permeabilized with 0.3% Triton-X in 1× PBS for 5 min at room temperature (21–22°C). Cells were washed twice with 1× PBS and incubated for 1 h in blocking solution (5% goat serum in 1× PBS). After 1 h, blocking solution was replaced with monoclonal mouse anti-microtubule-associated protein 2 (MAP2; Invitrogen, catalog #13-1500, neuronal marker) and polyclonal rabbit anti-GPER (Invitrogen, catalog #PA5-28647) antibodies diluted in blocking solution (1:200) and incubated overnight at 4°C. The primary antibodies were removed and cells were washed three times in 1× PBS for 10 min each. Fluorescently-tagged secondary antibodies Alexa Fluor 488 goat anti-mouse (ThermoFisher, catalog #A-11029) and Alexa Fluor 568 goat anti-rabbit (ThermoFisher, catalog #A-11011) diluted in blocking solution (1:500) were added and allowed to incubate for 1 h at room temperature. Secondary antibodies were then aspirated and cells were washed three times in 1× PBS then once in DI H_2_O. Cells were then mounted using Fluoroshield with DAPI (Sigma-Aldrich, catalog #F6507). Fluorescent images were taken using a confocal microscope (Leica TCS SP8), then processed and analyzed using ImageJ. The specificity of GPER and MAP2 antibodies was confirmed by staining neurons when either primary or secondary antibody was excluded from the incubation medium. Extended Data Figure 7-1*A* shows the results, which demonstrate that a fluorescence signal was only detected when both primary and secondary antibodies were included in the staining procedure. To verify whether neurons exhibited autofluorescence, the cells were excited with all lasers (568, 488, and 403 nm) of the confocal microscope and no autofluorescence was observed (data not shown).

### Western blotting

For immunoblotting, bilateral hippocampi and cortices were collected from individual brains of E18 embryos (*n* = 10 from three independent preparations). Tissues were treated with Laemmli sample buffer (Bio-Rad, catalog #1610611) containing 350 mm DTT (Bio-Rad, catalog #1610747) and run on a precast MES-SDS gel (NuPage, catalog #NP0323BOX) in a Novex Mini-Cell device (Invitrogen, catalog #EI0001). Bands were transfered to a 0.45 μm nitrocellulose membrane (Bio-Rad, catalog #1620115) in a Mini Protean Tetra System (Bio-Rad, catalog #1658004). Membranes were blotted using primary antibodies for GPER (Invitrogen, catalog #PA5-28647) and glyceraldehyde 3-phosphate dehydrogenase (GAPDH; CST, catalog #2118S, as loading control) at a 1:1000 dilution. A goat anti-rabbit secondary antibody (Invitrogen, catalog #31460) at a 1:5000 dilution was used for visualization. Western blot data were captured using an imager (ThermoFisher, iBright FL1000) after incubating the membranes in Pierce substrate (ThermoFisher, catalog #32106). The densitometric analysis was calculated using the area under the curve of peak intensity using ImageJ (NIH). Western blot experiments reveal two bands: a stronger band below 53 kDa (∼50 kDa) and a weaker band at ∼42 kDa. The two bands were only detected when both primary and secondary antibodies were used and no band was detected when excluding either the primary or the secondary antibody (Extended Data Fig. 7-1*B*). These data further confirmed the specificity of the GPER antibody used for this study.

### cDNA synthesis and real-time qPCR (RT-qPCR)

To assess GPER transcripts in cultures, RT-qPCR on the cDNA of RNA samples extracted from hippocampal and cortical cultures at 72 HIC was conducted. The synthesis of cDNA was performed using SuperScript IV VILO Master Mix (Invitrogen, catalog #11766050) following the manufacturer’s instructions. The subsequent RT-qPCR was performed on a QuantStudio 5 Real-Time PCR System (ThermoFisher, catalog #A28133) using SYBR Green PCR Master Mix (Applied Biosystems, catalog #4309155). The primers used were: Gper1 forward: CATGCCTACCCCTTGACAGG, reverse: TGGTATGACTGCCTTGAGCG. For normalization, the housekeeping gene (control) Gapdh was used, with primer sequences: forward: TCAACGGCACAGTCAAGGC, reverse: AGGGATGATGTTCTGGGCTG.

### RNA extraction, sequencing, and analysis

To investigate GPER’s effect on the neuron transcriptome, neurons were plated at a density of 500–600 cells/mm^2^ in 24-well plates (Becton Dickinson) and treated with either 0.1% DMSO (vehicle), E2 (100 nm), or G-1 (100 nm, selective GPER agonist) for 72 h. Subsequently, RNeasy Mini kit (QIAGEN, catalog #74104) was used for RNA extraction according to the manufacturer’s instructions. The quality and quantity were measured using NanoDrop (ThermoFisher Scientific).

For RNA sequencing, RNA samples were sent to Novogene (Novogene Corporation Inc.) and the sequencing was performed via Illumina NovaSeq 6000 platform based on the mechanism of SBS (sequencing by synthesis). For significance, a *p*-value cutoff of *p *<* *0.005 was used (Benjamini and Hochberg’s adjusted *p*-value). Unsupervised hierarchical clustering was performed with R Studio (Rstudio, 2015) using Pearson’s correlation distance matrix. Venn diagrams and correlation plots were generated using R Studio. Enrichment analysis was performed using gProfiler (Raudvere et al., 2019). The raw sequencing data generated in this study have been submitted to the NCBI BioProject database under accession number PRJNA759869.

### Chemicals

U73122 and U73343 were obtained from Tocris. G-1, G-15, and E2 were developed in Chris Arnatt’s lab. All other chemicals and cell culture reagents were obtained from Sigma-Aldrich or ThermoFisher unless stated otherwise.

### Statistical analysis

All statistics were conducted in R (Team, 2017) using RStudio (Rstudio, 2015) or GraphPad Prism 9 (GraphPad Software, San Diego, California USA). For transcriptomic data analysis, general code and packages used not stated in specific experiments include checking for packages and installing them, tidyverse (Wickham, 2017), data.table (Dowle and Srinivasan, 2019), emmeans (Lenth, 2019), multcomp (Hothorn et al., 2019), multicompview (Graves et al., 2015), plyr (Wickham, 2016), dplyr (Wickham et al., 2019), DescTools (Signorell et al., 2019), readxl (Wickham and Bryan, 2019), knitr (Xie, 2019), VennDiagram (Chen and Boutros, 2011), pheatmap (Kolde, 2019), and ggrepel (Slowikowski, 2020). For neurite outgrowth assay, a three-way ANOVA (*F*) test comparing type (cortical vs hippocampal), treatment, and time period followed by Tukey’s *post hoc* test was conducted to determine significance. For other experiments, data were statistically analyzed using Student’s *t* test or one-way ANOVA, followed by Tukey’s *post hoc* test to determine significant differences between treatments and/or cell types. For samples in neurite outgrowth and firing activity studies, which followed non-normal (Gaussian) distribution revealed by the Anderson–Darling, D’Agostino and Pearson, Shapiro–Wilk, and Kolmogorov–Smirnov tests, we also performed nonparametric Kruskal–Wallis (H) test followed by Dunn’s multiple comparisons *post hoc* test to confirm our conclusions. Values were considered statistically significant at the level of *p *<* *0.05. Because of the large datasets in our study and individual points become cluttered and difficult to read in most cases, the data are presented in figures as mean ± SEM. Each experiment was replicated a minimum of three times; the actual *F*_(dfs)_, *n*, and *p* values were provided in the text or figure legends.

## Results

### GPER agonists enhance neurite outgrowth in rat E18 hippocampal but not cortical neurons

**Figure 1.**
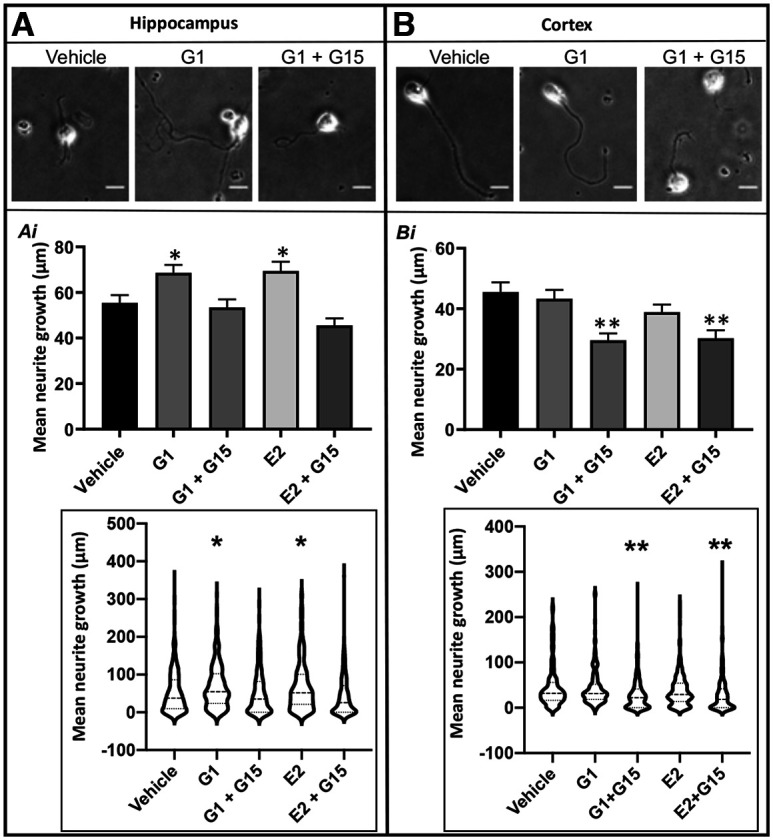
GPER activation increases neurite outgrowth in hippocampal but not cortical neurons. Selected images and results of hippocampal (***A***) and cortical (***B***) neurite outgrowth after 72 HIC. ***Ai***, Hippocampal neurons showed an increase in outgrowth compared with vehicle when GPER is activated with the selective agonist G-1 as well as with the nonspecific agonist E2. This effect was inhibited by the GPER-specific antagonist G-15. ***Bi***, Cortical neurons showed no significant effect of GPER activation on neurite outgrowth using either G-1 or E2 compared with vehicle control. Cortical neurite outgrowth was inhibited by blocking GPER activation using G-15. Significance was determined using two–way ANOVA followed by Dunnett’s test for multiple comparisons (hippocampal, *F*_(7,2527)_ = 10.73, *p* < 0.001, mean *n* = 223 cells; cortical, *F*_(7,1757)_ = 8.19, *p* < 0.001, mean *n* = 298 cells). Insets, Violin plot representation of the same datasets as shown in bar graphs. Statistical data of GPER activation/inactivation on hippocampal and cortical neurite outgrowth at all time points from 8 to 96 h are shown in Extended Data Figure 1-1. Data were from three independent experiments; **p <* *0.01 and *p <* *0.05, compared with vehicle. Scale bar: 20 μm. Treatments: G-1 (100 nm), E2 (100 nm), G-15 (10 nm).

The development of neuronal processes (neurites, including axons and dendrites) is the essential developmental event that allows for the proper formation of neuron-neuron connections (synapses) during early nervous system development and after nerve injury. We first sought to define the impact of GPER activation on neurite outgrowth of rat E18 hippocampal and cortical neurons. Specifically, hippocampi and cortices were dissected from rat E18 embryo brains and were enzymatically digested followed by gentle mechanical trituration. Neurons were cultured in the absence or presence of GPER agonists and/or antagonists and phase contrast and neurite tracing were performed to evaluate their effects (Fig. 1; Extended Data Fig. 1-1). Based on the selective binding profiles of GPER agonist G-1 and antagonist G-15 (see Materials and Methods) and physiological studies in the field (McCarthy, 2008; de Valdivia et al., 2017; Kumar and Foster, 2020), we initially tested the effects of G-1 alone at 100 nm and G-15 alone at 10 nm on the growth of cultured hippocampal and cortical neurons compared with neurons cultured in vehicle (0.1% DMSO). Our results showed that in hippocampal neurons G-1 alone significantly (*p* < 0.01) increased average neurite outgrowth (μm), while G-15 alone had no effect (*p* > 0.05) compared with vehicle (one-way ANOVA, *F*_(2,638)_ = 18.46, *p* < 0.01; Vehicle: 52.4 ± 3.1, *n* = 264 cells; G-1: 78.1± 4.4, *n* = 180 cells; G15: 45.2 ± 4.1, *n* = 197 cells). In contrast to hippocampal neurons, G-1 alone did not affect neurite outgrowth (*p* > 0.05) in cortical neurons, but interestingly, G-15 treatment alone led to significantly reduced growth of cortical neurons compared with their vehicle or G-1 treatment (one-way ANOVA, *F*_(2,245)_ = 11.6, *p* < 0.01; vehicle: 21.6 ± 2.9, *n* = 112 cells; G-1: 21.3 ± 4.0, *n* = 54 cells; G-15: 4.6 ± 1.5, *n* = 82). These initial experiments revealed intriguing, distinct effects of GPER activation or inactivation on neurite growth in these two different cell types.

10.1523/ENEURO.0475-21.2022.f1-1 Extended Data Figure 1-1 GPER agonists increase neurite outgrowth in hippocampal but not cortical neurons. GPER activation by a selective agonist, G-1, and nonselective agonist, E2, increases neurite outgrowth in hippocampal neurons at multiple time points as specified as HIC, and blocking GPER activation with a selective antagonist, G-15, inhibits this effect. In cortical neurons activation of GPER does not appear to enhance outgrowth except at 96 HIC, but blocking GPER activation inhibited neurite outgrowth throughout; *p <* *0.05 versus vehicle, **p <* *0.01 versus vehicle. Download Figure 1-1, TIF file.

To further test the role of GPER activation by agonists on neuronal outgrowth of E18 hippocampal and cortical neurons at different early developing time points, neurons were cultured in vehicle (0.1% DMSO), E2 (100 nm), and G-1 (100 nm), with or without the selective GPER antagonist G-15 (10 nm) for 4 d. Phase-contrast images were taken every day for 4 d, and the ImageJ plugin NeuronJ was employed to measure neurite outgrowth per cell (Schindelin et al., 2012; Imaninezhad et al., 2018; Pemberton et al., 2018; see Materials and Methods). Figure 1 shows the representative images of hippocampal and cortical neurons in vehicle and drug-treated conditions at 72 HIC. Statistical data of GPER activation/inactivation on hippocampal and cortical neurite outgrowth at all time points from 8–96 h are shown in Extended Data Figure 1-1. Data were collected from cells cultured in three independent experiments with three replications per treatment group at each time point. Overall, a three-way ANOVA comparing type (cortical vs hippocampal), dose/treatment, and time period showed significant differences at each level (*F*_(1,24269)_ = 346.28, *F*_(7,24269)_ = 38.78, *F*_(4,24269)_ = 2710 respectively; all *p *<* *0.001) as well as significance between each interaction (type:treatment *F*_(7,24269)_ = 12.812, type:time *F*_(4,24269)_ = 151.62, treatment:time *F*_(28,24269)_ = 14.78, type:treatment:time *F*_(28,24269)_ = 5.78; all *p *<* *0.001). *Post hoc* tests for individual groups showed 8 HIC treatments had no significant difference between any groups compared with vehicle. At each time point after 8 HIC, both hippocampal and cortical neurons showed significant differences between different treatment groups based on one-way ANOVAs (Cortical, 20 HIC *F*_(7,2186)_ = 11.10, *p *<* *0.001; 48 HIC *F*_(7,2077)_ = 9.52, *p *<* *0.001; 72 HIC *F*_(7,1757)_ = 8.19, *p *<* *0.001; 96 HIC *F*_(7,1764)_ = 24.15, *p *<* *0.001: Hippocampal – 20 HIC *F*_(7,3029)_ = 6.60, *p *<* *0.001, 48 HIC *F*_(7,2397)_ = 6.65, *p *<* *0.001; 72 HIC *F*_(7,2527)_ = 10.73, *p *<* *0.001; 96 HIC *F*_(7,1764)_ = 14.58, *p *<* *0.001). Specifically, in hippocampal neurons G-1 significantly enhanced neurite outgrowth at 48 and 72 HIC and this effect was attenuated by blocking GPER activation with antagonist G-15, indicating the involvement of GPER activity (72 HIC comparisons vs vehicle; Fig. 1*Ai*,*Bi*, insets). E2 also enhanced neurite outgrowth at 72 and 96 HIC which was blocked by G-15, further implicating the involvement of GPER in estrogen action in hippocampal neurons. Interestingly, in cortical neurons, activation of GPER with G-1 or E2 did not significantly increase neurite outgrowth except at 96 HIC. Additionally, inhibition of GPER by G-15 either in the absence or presence of GPER agonists significantly inhibited neurite outgrowth in cortical neurons at 20–96 HIC; in hippocampal neurons this inhibition was only seen at 20 HIC. Because our normality tests with the Anderson–Darling, D’Agostino and Pearson, Shapiro–Wilk, and Kolmogorov–Smirnov tests suggested that the neurite outgrowth data followed a non-normal (Gaussian) distribution pattern, we also performed nonparametric Kruskal–Wallis test followed by *post hoc* Dunn’s multiple comparisons test to determine whether our conclusions could be changed. We found that the significance between different treatment groups compared with vehicle mirrored that of the Tukey test following the parametric ANOVA analyses. For example, the nonparametric Kruskal–Wallis test followed by *post hoc* Dunn’s test also showed that 8 HIC treatments had no significant difference between any groups compared with vehicle in hippocampal neurons (H_(4)_ = 7.888, *p *=* *0.09), although there was a significant difference in cortical neurons (H_(4)_ = 42.01, *p *<* *0.0001). Consistently, at each time point after 8 HIC, both hippocampal and cortical neurons showed significant differences between different treatment groups (Cortical, 20 HIC H_(4)_ = 63.06, *p *<* *0.0001; 48 HIC H_(4)_ = 104.5, *p *<* *0.0001; 72 HIC H_(4)_ = 56.6, *p *<* *0.0001; 96 HIC H_(4)_ = 122.6, *p *<* *0.0001: Hippocampal, 20 HIC H_(4)_ = 59.19, *p *<* *0.0001, 48 HIC H_(4)_ = 22.8, *p *<* *0.0001; 72 HIC H_(4)_ = 57.17, *p *<* *0.0001; 96 HIC H_(4)_ = 53.44, *p *<* *0.0001). It is important to note here that the nonparametric Kruskal–Wallis test not only agreed well with our initial parametric ANOVA test for detecting significant differences among treatment groups, but also often revealed more robust significance levels (e.g., *p *<* *0.0001 vs *p *<* *0.001 shown by parametric ANOVA test) in many of our samples, further confirming our conclusions. Together, these data suggest that pharmacological activation or inactivation of GPER induced distinct effects on primary E18 rat hippocampal and cortical neurons. GPER likely plays stimulatory roles in neurite outgrowth of hippocampal neurons, but the effects of GPER activation/inactivation on cortical neurons varied either having no effects or inhibitory effects.

### GPER agonists increase neuronal firing activity in hippocampal and cortical neurons with a more potent effect on hippocampal neurons

**Figure 2.**
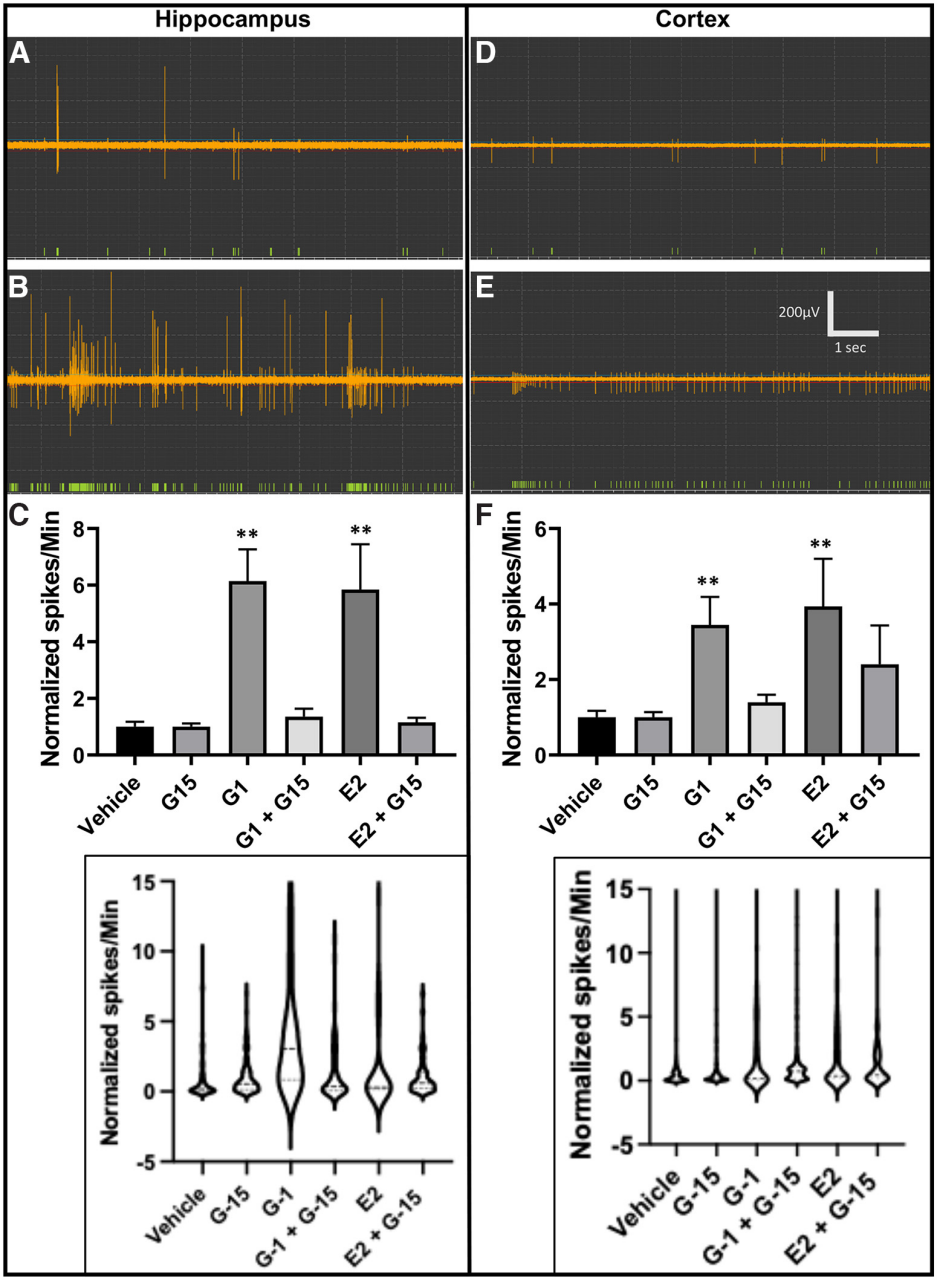
GPER activation increases neuronal firing activity in hippocampal and cortical neurons. Representative readings from hippocampal (***A***, ***B***) and cortical (***D***, ***E***) neurons show a much lower frequency of spikes (green bars) before the introduction of G-1 (***A***, ***D***) compared with the number of spikes after GPER activation using G-1 (***B***, ***E***). Statistical data (***C***, ***F***) show that both the selective GPER agonist G-1 and nonspecific agonist E2 significantly increase the frequency of neuronal firing activity in hippocampal and cortical neurons cultured for 14 d (values normalized to the response before introduction of the drug as a value of 1). This significant increase in spiking activity from both G-1 and E2 is inhibited by the pretreatment with GPER-specific antagonist G-15 (***C***, ***F***). Hippocampus one-way ANOVA, G-1, E2 *F* = 14.28, *p *<* *0.001, *n* =* *60; cortex one-way ANOVA, G-1, E2 *F* = 4.66, *p *<* *0.001, *n *=* *180; **p < 0.01, compared with vehicle. Treatments: G-1 (100 nm), E2 (100 nm), G-15 (10 nm). Insets, Violin plot representation of the same datasets as shown in bar graphs.

It is widely accepted that neurons use electrical activity, also referred to as nerve impulses and action potentials, for neuronal signaling. This neuronal activity has been implicated in a myriad of processes including neural development, synaptic transmission, and synaptic plasticity (Spitzer, 2006; Borodinsky and Belgacem, 2016; Luhmann et al., 2016; A.M. Walter et al., 2018; Kato and Wake, 2019). Because of the observed differential neurite outgrowth in hippocampal and cortical neurons in response to GPER activation, we hypothesized that GPER stimulation results in differences in neuronal electrical activity in hippocampal and cortical neurons. To test this hypothesis, noninvasive MEA (multichannel) neurochip interface technology was used to monitor neuronal firing activity from a group of developing neurons before and after exposure to GPER agonists G-1 or E2. Subsequent studies were performed to examine whether neuronal firing activity could be blocked with the GPER antagonist G-15. Overall, our neurochip recording experiments revealed that there was no significant difference in the baseline (before the treatments) frequency of spiking between hippocampal and cortical cultures (unpaired Student’s *t* test, *p* = 0.9; Hippocampal, 982.3 ± 195, *n* = 120 electrodes; Cortical, 914.9 ± 262.4, *n* = 480 electrodes), but the frequency of neuronal activity was significantly increased in both hippocampal and cortical neurons after G1 or E2 treatments, with a more potent effect observed in hippocampal neurons (Fig. 2). The representative trace in Figure 2*A* shows that hippocampal neurons at rest were spontaneously active (green bars represent program detected spikes). After the application of 100 nm G-1 (Fig. 2*B*), the firing frequency (spikes/min) was greatly increased. The statistical data for hippocampal cultures (Fig. 2*C*) show that both G-1 (one-way ANOVA, *F*_(5,474)_ = 14.28, *n* =* *60 electrodes for each condition from 3 independent experiments; Tukey’s *post hoc* analysis, *p *<* *0.001) and E2 (*p *<* *0.001) significantly increased the neuronal firing activity compared with the control, and the increase in neuronal firing was prevented by G-15 pretreatments (G-1 vs G-1 + G-15; *p *<* *0.001, E2 vs E2 + G-15; *p *<* *0.001). Similar to hippocampal neurons, cortical neurons (Fig. 2*D–F*) exhibited a significant increase in the frequency of neuronal firing in response to G-1 (one-way ANOVA, *F*_(5,1552)_ = 4.66; Tukey’s *post hoc* analysis, *p *<* *0.001, *n* =* *180) and E2 (*p *<* *0.001, *n *=* *180); however, the magnitude of neuronal activity between the hippocampal and cortical neurons differed (4-fold vs 2-fold increase in firing by G-1; Fig. 2*C*,*F*). Additionally, in cortical neurons, attenuation of neuronal firing with G-15 was present but not statistically significant (*p *=* *0.17, *n *=* *180 for G-1 + G-15; *p *=* *0.88, *n *=* *60 for E2 + G-15). Similar to the neurite outgrowth data, the normality tests show that these samples followed a non-normal distribution. We then performed the Kruskal–Wallis test followed by *post hoc* Dunn’s multiple comparisons test to further confirm our conclusions. Again, the Kruskal–Wallis test mirrored that of parametric ANOVA test results in most comparisons between groups, although a more robust significance level was once again observed in many comparisons. For example, G1 and E2 were found to significantly increase neuronal firing activity in hippocampal (Kruskal–Wallis test, H_(5)_ = 67.13, *p *<* *0.0001) and cortical (H_(5)_ = 66.11, *p *<* *0.0001) neurons. Dunn’s *post hoc* analysis showed that in hippocampal neurons, G1 significantly increased firing (vehicle vs G-1, *p *<* *0.0001) and the antagonist G15 alone did not affect neuronal activity (vehicle vs G-15, *p *>* *0.05), but significantly reduced G-1-induced increase in neuronal activity (G-1 vs G-1+G-15, *p* < 0.0001). In cortical neurons, G1 significantly increased firing (vehicle vs G-1, *p *<* *0.0001), and the antagonist G15 reduced G-1-induced increase in activity, but this reduction did not reach statistical significance level (G-1 vs G-1 + G-15, *p* > 0.05), which is consistent with the parametric ANOVA analysis results. Together, these results demonstrate the pivotal role of GPER in stimulating neuronal activity in hippocampal and cortical neurons although the magnitude of neuronal activity in response to GPER activation/inactivation differs between hippocampal and cortical neurons.

### GPER agonists increase Ca^2+^ activity in hippocampal but not cortical neurons

**Figure 3.**
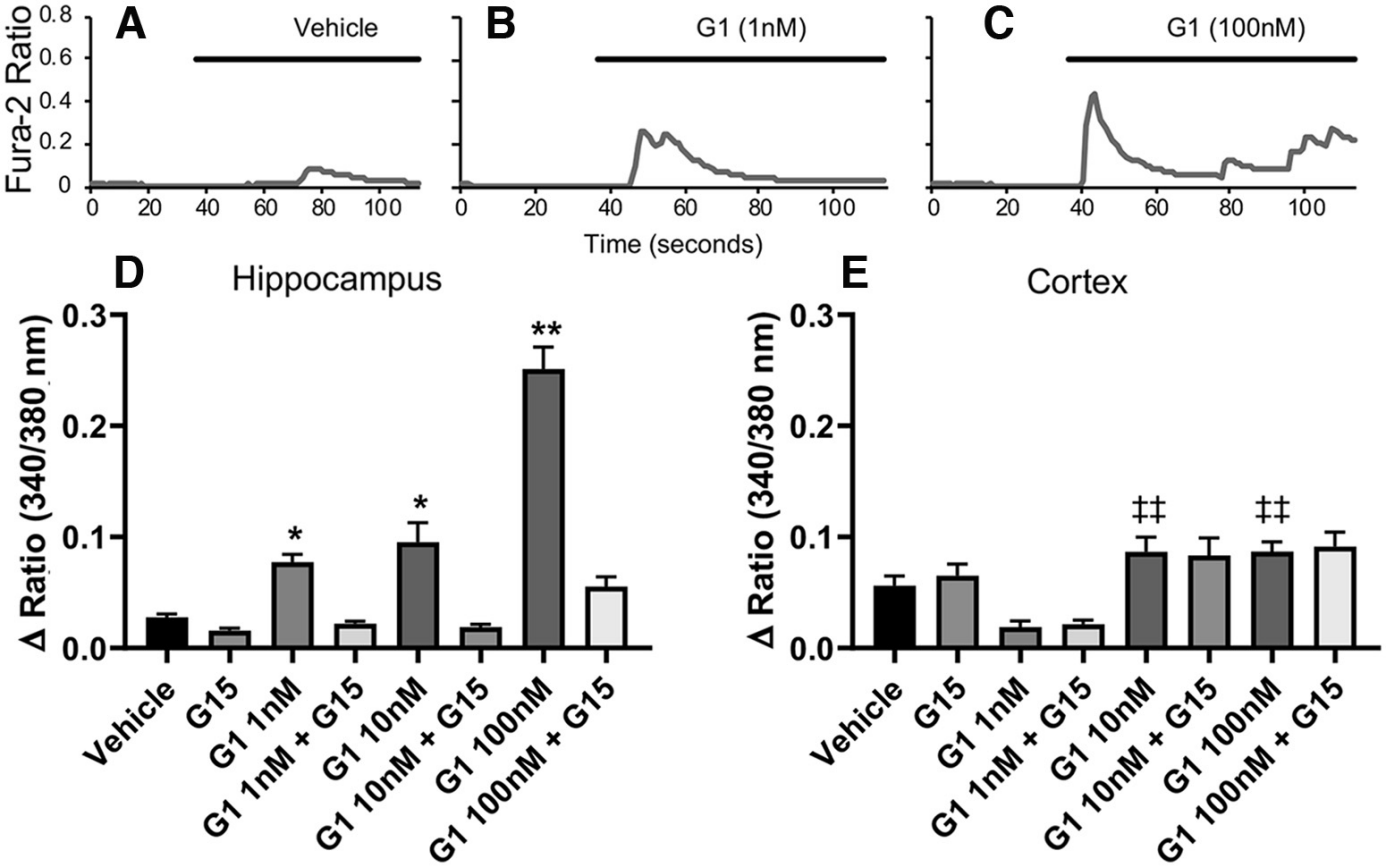
GPER activation increases cytosolic Ca^2+^ in hippocampal but not cortical neurons. Fura-2 ratiometric Ca^2+^ traces of hippocampal neurons (***A–C***) showed a dose-dependent increase in Ca^2+^ response to GPER-selective agonist G-1. Vehicle (***A***) showed little to no Ca^2+^ response while 1 nm G-1 (***B***) showed a significant increase in Ca^2+^ response, and 100 nm G-1 (***C***) showed the greatest increase in Ca^2+^. ***D***, Mean values of peak amplitude of Ca^2^**^+^** increase in hippocampal neurons (one-way ANOVA *F* = 80.97, *n *=* *92, Turkey’s *post hoc* analysis 1 nm G-1 *p *=* *0.036, 10 nm G-1 *p *=* *0.031, 100 nm G-1 *p *<* *0.001). These effects were blocked by 10 nM G-15. ***E***, In cortical neurons, GPER activation lowered levels of Ca^2+^ at 1 nm G-1 while increasing Ca^2+^ at higher concentrations of G-1, although these did not reach significance (G-1 1 nm *p *=* *0.20, G-1 10 nm *p *=* *0.64, G-1 100 nm *p *=* *0.45). These effects were not blocked by G-15; *p <* *0.05, **p <* *0.01, compared with vehicle; ‡‡*p *<* *0.01, compared with G-1 1 nm.

Among ions in flux during neuronal firing is Ca^2+^, a key second messenger commonly involved in coupling external stimuli and neuronal firing to cytosolic signaling to regulate a variety of neurodevelopmental processes (Rosenberg and Spitzer, 2011; Leclerc et al., 2012). For this reason, we next tested whether Ca^2+^ signaling in response to GPER activation may be altered between hippocampal and cortical neurons. To test this, we conducted fura-2 AM ratiometric Ca^2+^ imaging experiments on both hippocampal and cortical neurons. Our Ca^2+^ imaging data revealed that in hippocampal neurons, GPER activation by G-1 significantly (one-way ANOVA, *F*_(8,1166)_ = 80.97, mean *n *=* *92; Tukey’s *post hoc* analysis, *p *<* *0.001) increased the peak amplitude of Ca^2+^ signals in a dose-dependent manner (1 nm G-1, *p *=* *0.036; 10 nm G-1, *p *=* *0.031; and 100 nm G-1, *p *<* *0.001; Fig. 3*A–D*) compared with vehicle. The observed increase in Ca^2+^ in response to G-1 and E2 was abolished by pretreatment with the selective GPER antagonist, G-15, for 30 min before treatment. In contrast, cortical neurons did not exhibit significant change in Ca^2+^ in response to GPER activation as compared with vehicle (Fig. 3*E*; 1 nm G-1, *p *=* *0.203; 10 nm G-1, *p *=* *0.641; 100 nm G-1, *p *=* *0.451), although there were some significant differences between concentrations of G-1 (one-way ANOVA, *F*_(7,1488)_ = 9.46, mean *n *=* *182; Tukey’s *post hoc* analysis, *p *<* *0.001; G-1 1 nm vs G-1 10 nm *p *<* *0.001, vs G-1 100 nm *p *<* *0.001). These results show that although GPER activation increased the frequency of neuronal firing activity in cortical neurons, there was no significant change in intracellular Ca^2+^ levels. The absence of Ca^2+^ signaling may contribute to the lack of neurite outgrowth observed in cortical neurons in response to GPER activation. By this same logic, the correlation between electrical activity and Ca^2+^ signaling in response to G-1 and/or E2 in hippocampal neurons may contribute to the GPER-induced neurite outgrowth in these neurons.

### GPER agonist-mediated Ca^2+^ rise in hippocampal neurons involves extracellular Ca^2+^ entry via Ca^2+^ channels and internal Ca^2+^ release from phospholipase C (PLC)-inositol trisphosphate (IP_3_) Ca^2+^ stores

**Figure 4.**
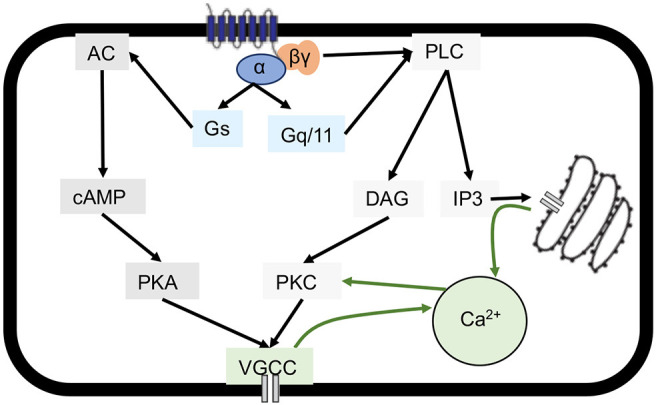
Representative model of common GPCR signaling pathways leading to an increase in cytosolic Ca^2+^. Different GPCR pathways can converge to an increase in intracellular Ca^2+^ levels. G_s_ subunit coupling leads to the activation of AC that increases cAMP levels and promotes the activity of PKA. PKA leads to the opening of VGCCs and, therefore, extracellular Ca^2+^ entry via VGCCs. G_q/11_ subunit coupling leads to the activation of PLC which catalyzes the production of DAG and IP_3_. IP_3_ can then bind to IP_3_ receptor-gated Ca^2+^ channels present on the endoplasmic reticulum, leading to Ca^2+^ release from intracellular stores. The subsequent rise in intracellular Ca^2+^, together with DAG, activates PKC which causes the opening of VGCCs. The βγ dimer of G-proteins has also been shown to activate PLC or directly regulate VGCCs and lead to an increase in cytosolic Ca^2+^.

**Figure 5.**
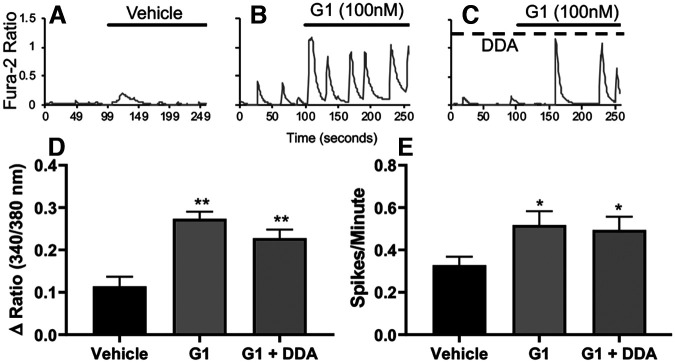
GPER- induced Ca^2+^ rise is not dependent on cAMP production. ***A–C***, Representative traces of fura-2 ratiometric Ca^2+^ imaging for vehicle (***A***), G1 alone (***B***), and G1 with selective inhibitor of AC DDA (10 μm; ***C***). Statistical data demonstrate that G-1 still significantly increased both the mean values of peak amplitude of Ca^2^**^+^** increase (***D***) and the mean frequency of Ca^2+^ spiking activity (***E***) when cAMP production is inhibited by DDA (G-1 *p *<* *0.001, *n *=* *131, DDA + G-1 *p *<* *0.001, *n *=* *138, vehicle *n* = 90); *p < 0.05 and **p < 0.01, compared with vehicle.

To gain further insight into the signaling pathway involved in G-1-induced Ca^2+^ rise, the goal of subsequent studies was to understand the source of Ca^2+^ in hippocampal neurons. Intracellular Ca^2+^ mobilization primarily originates from either the influx of extracellular Ca^2+^ or the release of Ca^2+^ from intracellular Ca^2+^ stores. Canonical GPCR Ca^2+^ signaling is illustrated by a schematic drawing in Figure 4. The canonical method of GPCR Ca^2+^ mobilization occurs through the coupling to the G_q/11-_PLC-IP_3_-Ca^2+^ channels on the endoplasmic reticulum that are gated by IP_3_ receptor. An alternative signaling pathway for Ca^2+^ mobilization can occur through coupling to G_s_-adenylate cyclase (AC)-cAMP-protein kinase A (PKA)-voltage-gated Ca^2+^ channels (VGCCs). In addition to these pathways, PLC-induced production of DAG can subsequently activate protein kinase C (PKC), which in turn leads to the opening of VGCC and an increase in intracellular Ca^2+^. Lastly, the βγ dimer of G-proteins has also been shown to activate PLC or directly modulate VGCCs for Ca^2+^ mobilization (De Waard et al., 1997; Uezono et al., 2004; Smrcka, 2008; Proft and Weiss, 2015). The potential involvement of store-operated Ca^2+^ channels (SOCs) which is activated by the depletion of Ca^2+^ from the endoplasmic reticulum (Prakriya and Lewis, 2015) is not included in the schematic drawing. Because GPER has been shown to increase the level of cAMP in hippocampal neurons (Evans et al., 2016) and both cAMP and the downstream effector PKA have been shown to regulate Ca^2+^ signaling (Taylor, 2017), we first sought to determine the involvement of the G_s_/cAMP/PKA pathway in G-1-induced Ca^2+^ rise in hippocampal neurons using a selective inhibitor of adenylyl cyclase [AC; 2′,3′ dideoxyadenosine (DDA), 10 μm; Leavitt et al., 1987; Amendola et al., 2012] to inhibit cAMP production. Specifically, hippocampal neurons were pretreated with vehicle or DDA alone for at least 30 min before exposure to G-1. Figure 5 shows the δ (Δ) level of increase in mean peak amplitude or frequency of Ca^2+^ spikes by G-1 over basal Ca^2+^ level in vehicle or DDA alone (data not shown). The results (Fig. 5) showed that 100 nm of G-1 (one-way ANOVA, *F*_(4,473)_ = 22.38; Tukey’s *post hoc* analysis, *p *<* *0.001, *n *=* *131 cells) significantly increased the peak amplitude of Ca^2+^ spikes compared with vehicle and that pretreatment with DDA failed to prevent the increase in Ca^2+^ amplitude because of G-1 (*p *<* *0.001, *n *=* *138 cells) compared with DDA alone (*n *=* *90 cells). Moreover, both G-1 and G-1 plus DDA significantly increased the frequency of Ca^2+^ spikes (G-1 alone, *p *<* *0.05, *n *=* *40 neurons; G-1 with DDA, *p *<* *0.05, *n *=* *43) compared with vehicle (*n *=* *72). These data were from four replications with three trials. These data indicate that the G_s_/cAMP/PKA may not be directly involved in GPER-mediated regulation of Ca^2+^ in hippocampal neurons.

**Figure 6.**
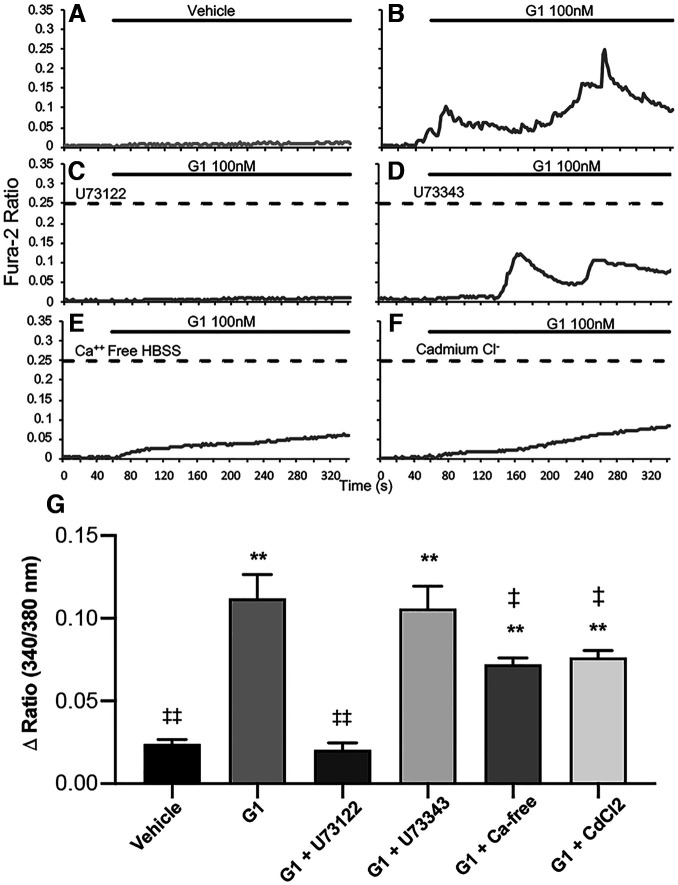
GPER-induced Ca^2+^ rise in hippocampal neurons involves both extracellular Ca^2+^ entry via VGCCs and PLC-mediated internal Ca^2+^store release. ***A–F***, Representative traces of ratiometric Ca^2+^ imaging in vehicle (***A***), G-1 alone (***B***), G-1 plus PLC inhibitor (U73122, 10 μM; ***C***), G-1 plus the inactive analog of the PLC inhibitor (U73343, 10 μM; ***D***), G-1 in Ca^2^**^+^**-free buffer (***E***), and G-1 plus 100 μM cadmium Cl- (CdCl_2_), a nonspecific blocker of VGCCs (***F***). ***G***, Statistical data show that GPER-induced increase in intracellular Ca^2+^ is blocked fully by inhibition of IP_3_ production (U73122 *p *=* *0.999 vs vehicle, *p *<* *0.01 vs G-1), but only partially, yet significantly, inhibited by removal of external Ca^2+^ ions (Ca^2+^-free; *p *<* *0.05 vs G-1) or by blocking of Ca^2+^ entry with CdCl_2_ (*p *<* *0.05 vs G-1); **p <* *0.01 versus vehicle, ‡*p *<* *0.05 versus G-1, ‡‡*p *<* *0.01 versus G-1.

We then examined whether G-1-induced intracellular Ca^2+^ involves the activation of PLC-IP_3_ pathway in hippocampal neurons. To this end, neurons were pretreated with a selective PLC inhibitor, U73122 (10 μm), and its inactive form, U73343 (10 μm; Jin et al., 1994; Takenouchi et al., 2005) for at least 30 min before exposure to G-1. Our data showed that G-1-induced Ca^2+^ increase was abolished with the PLC inhibitor U73122 (one-way ANOVA, *F*_(5,271)_ = 22.39, Tukey *post hoc* analysis; *p *=* *0.999 vs vehicle control; *p *<* *0.001 vs G-1 alone), while the Ca^2+^ increase remained using the inactive analog U73343 (*p *<* *0.001 vs vehicle; *p *=* *0.996 vs G-1 alone; Fig. 6*A–D*,*G*). We also found that chelerythrine chloride, a selective inhibitor of PKC, the downstream effector of PLC-diacylglycerol (DAG)-Ca^2+^, did not affect the ability of G-1 to increase Ca^2+^ in hippocampal neurons. Specifically, the change in Ca^2+^ ratio in G-1 alone was 0.46 ± 0.04 (*n* = 54) and in G-1 + chelerythrine chloride was 0.48 ± 0.02 (one-way ANOVA, *F*_(4,473)_ = 22.38, Tukey’s *post hoc* analysis*; p *>* *0.05 vs G-1 alone, *n* = 58; data not shown). These data indicate that PKC is not responsible for independently activating Ca^2+^ channels. Based on the findings with the PLC inhibitor, U73122, and the PKC inhibitor, chelerythrine chloride, it appears that at least some of the Ca^2+^ mobilization in response to GPER activation occurs through the PLC-IP_3_ signaling cascade.

Pertaining to GPER, evidence also suggests that the L-type Ca^2+^ channel Cav1.3 is responsible for estrogen-stimulated Ca^2+^ influx in endometrial cancer and vascular smooth muscle cells (Hao et al., 2015; Ji et al., 2016). Therefore, we next also examined the involvement of extracellular Ca^2+^ entry in response to GPER activation in hippocampal neurons. To do this, we used either a Ca^2+^-free buffer (Fig. 6*E*) or added a commonly used nonselective VGCC blocker, cadmium chloride (CdCl_2_; Fig. 6*F*; Dascal et al., 1986; Tredway et al., 1999; Chin et al., 2003; Martínez Damonte et al., 2018; Lee et al., 2022) to block extracellular Ca^2+^ entry via VGCCs. We found that in assays with Ca^2+^ supplemented buffer, G-1 increased the intracellular Ca^2+^ level in hippocampal neurons compared with vehicle (Fig. 6*E–G*), and this rise in Ca^2+^ mobilization in response to GPER activation by G-1 was significantly attenuated in a Ca^2+^-free buffer or with CdCl_2_ (100 μm; one-way ANOVA, *F*_(5,271)_ = 22.39, Tukey’s *post hoc* analysis; *p *<* *0.05 vs G-1 for both). These data indicate that G-1-induced Ca^2+^ rise involves extracellular Ca^2+^ entry likely via VGCCs. It is important to note that Ca^2+^ rise in response to G-1 in the absence of external Ca^2+^ or with CdCl_2_ still exhibited significant Ca^2+^ rise compared with vehicle (*p *<* *0.001 vs vehicle control for both scenarios), further indicating that internal Ca^2+^ store release also contributes significantly to GPER-mediated Ca^2+^ rise in hippocampal neurons.

Together, our Ca^2+^ imaging experiments showed that G-1 increases cytosolic Ca^2+^ in rat hippocampal, but not cortical, neurons. This rise in hippocampal intracellular Ca^2+^ involves both internal Ca^2+^ release mediated by the PLC-IP_3_ pathway and external Ca^2+^ entry potentially via VGCCs. The interplay of Ca^2+^ from both sources is essential for the maintenance of spontaneous Ca^2+^ spiking activity (oscillations) and the G-1-mediated increase in intracellular Ca^2+^ in hippocampal neurons. The different effectiveness of GPER on Ca^2+^ signaling in rat E18 hippocampal versus cortical neurons may partially contribute to its distinct effects on neurite outgrowth in cultured hippocampal and cortical neurons. Next, we sought to determine the molecular mechanisms that might contribute to the distinct effects of GPER-medicated neural outgrowth of hippocampal and cortical neurons.

### GPER expression in E18 hippocampal and cortical primary cultures and tissues

**Figure 7.**
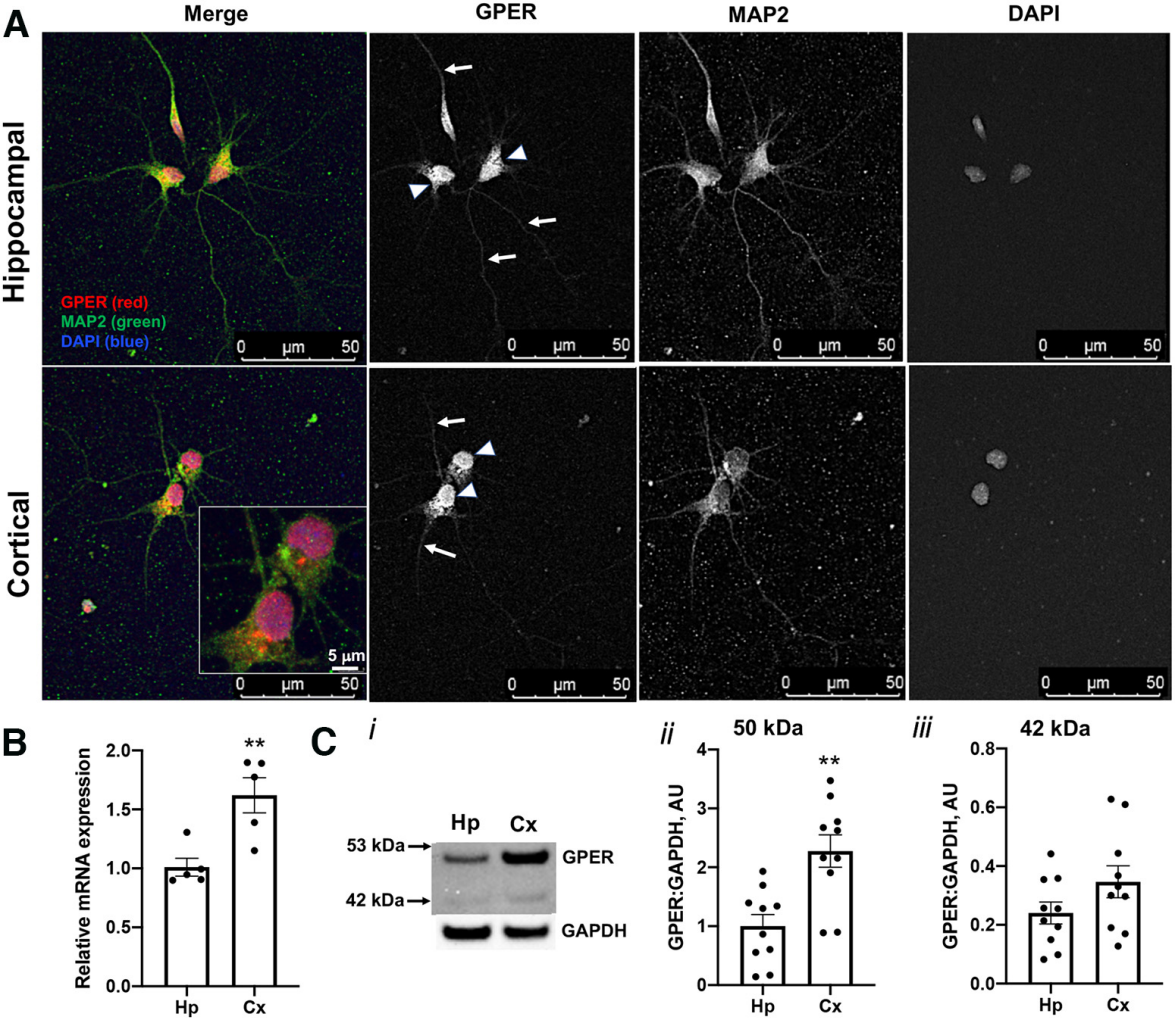
Studies of GPER expression in hippocampal and cortical cultures and tissues. GPER expression and localization in cultured hippocampal and cortical neurons 72 HIC were measured using immunofluorescent and confocal microscopy techniques. ***A***, Representative fluorescence images showing the localization of GPER (red) in cell bodies (arrowheads) and neurites (arrows). Cell nuclei are labeled with DAPI (blue) and neurites are labeled with MAP2 (green). ***B***, RT-qPCR measurement of the relative GPER mRNA level in samples derived from hippocampal and cortical culture at 72 HIC shows a significantly (unpaired Student’s *t* test, *p* < 0.01, *n* = 5 replicates) higher GPER mRNA level in cortical than hippocampal cultures. ***C***, Western blot measurements of GPER protein expression in *ex vivo* hippocampal and cortical tissues from individual E18 rat brains (*n* = 10) reveals two protein species with mass sizes of ∼50 and 42 kDa (***i***). Statistical analysis shows that expression of GPER ∼50 kDa is significantly higher in cortical than in hippocampal tissues (***ii***), while GPER ∼42 kDa is slightly, but not significantly, higher in cortical than in hippocampal tissues (***iii***). Unpaired Student’s *t* test; **p < 0.01. The specificity of the GPER antibody used for this study was validated by immunocytochemistry and Western blotting. The validation results are shown in Extended Data Figure 7-1.

We hypothesized that a richer expression of GPER in hippocampal neurons may account for the stronger effects of GPER in hippocampal neuronal growth and activity. We first determined whether differential expression or distribution of GPER occurred in cultured rat E18 hippocampal and cortical neurons. Hippocampal and cortical neurons were cultured, fixed at 72 HIC, and fluorescently stained with antibodies against GPER (red; Invitrogen, catalog #PA5-28 647) and MAP2 (neuronal dendritic marker, green; Invitrogen, catalog #13-1500) with DAPI (blue) labeling nuclei. The results show that GPER is abundantly expressed in both hippocampal and cortical neurons. While in both cell types GPER expression is strong in the cell bodies (indicated by arrowheads), it is also weakly expressed in the neurites (Fig. 7*A*, arrows). Subcellularly, GPER is largely located in the cytosol as well as in the nuclear area of both hippocampal and cortical neurons. Overall, there is no apparent difference in GPER’s localization at distinct neuronal compartments (soma vs neurite) or subcellular structures in hippocampal and cortical neurons. Next, we tested whether the expression level of GPER differs between hippocampal and cortical neurons by measuring GPER mRNA via RT-qPCR and GPER protein expression via Western blotting. For qPCR, hippocampal and cortical neurons were cultured for 72 h and RNA was extracted for subsequent measurement of GPER transcripts. To our surprise, qPCR data (Fig. 7*B*) showed that GPER transcripts are significantly more abundant in cortical cultures than hippocampal cultures (*p* < 0.05, unpaired Student’s *t* test, *n* = 5 replications). This result contradicts our original postulation that a more robust physiological response to G-1 in hippocampal compared with cortical cultures corresponds to a greater GPER mRNA expression. We then wondered whether GPER protein level has higher expression in hippocampal than in cortical cells. Unfortunately, the small amount of proteins extracted from primary cultures did not yield clear and reliable bands of GPER proteins when assayed with Western blotting. We then performed a Western blotting using *ex vivo* tissues of rat E18 hippocampus and cortex. Specifically, bilateral hippocampus and cortex from individual E18 embryos were harvested, sonicated, and suspended in lysis buffer in preparation for Western blotting with antibodies to GPER (Invitrogen, catalog #PA5-28 647) and GAPDH (CST, catalog #2118S, as loading control). Western blotting detected two bands in both hippocampal and cortical tissues (Fig. 7*C*): one stronger band at ∼50 kDa and one weaker band at ∼42 kDa. The lower GPER mass species such as ∼42 kDa and higher band protein species were reported previously and were found to be caused by GPER N-glycosylation or nonglycosylation (de Valdivia et al., 2017, 2019). Interestingly, our data showed that the normalized ratio of GPER:GAPDH (AU) was significantly higher in the cortex (Cx) than hippocampus (Hp) for the 50 kDa; GPER at 42 kDa showed a higher expression in the cortex, but the level of difference was not statistically significant (*p* > 0.05, unpaired Student’s *t* test, *n* = 10 animals from three preparations). Overall, these experiments indicate that there is no apparent difference in GPER localization in hippocampal and cortical neurons, yet GPER mRNA level measured by qPCR in cortical cultures is significantly higher than in hippocampal cultures. Similarly, GPER protein level (specifically the ∼50 kDa) appears to be more abundant in E18 rat cortex than in the hippocampus. Taken together, these data did not support our hypothesis that the robust GPER stimulatory action in hippocampal neurite outgrowth, firing, and Ca^2+^ rise may depend on GPER being more heavily expressed in hippocampal neurons. These data instead indicate that the difference in physiological response by cell type may be because of other cellular mechanisms such as heterotrimeric G-protein association, cellular signaling, and specific molecular effector coupling. For this purpose, we performed RNA sequencing to explore the potential genes and pathways that might contribute to the distinct effects of GPER-mediated neuronal outgrowth of hippocampal and cortical neurons.

10.1523/ENEURO.0475-21.2022.f7-1 Extended Data Figure 7-1 Validation of antibody specificity by immunofluorescent and Western blot experiments. ***A***, To verify the specificity of GPER (red) and MAP2 (green, neuronal marker) antibodies used in the fluorescence studies, neurons were fixed and immunostained in incubation medium with no antibody (***i***), only primary antibodies for GPER and MAP2 (***ii***), only secondary antibodies for Alexa Fluor 568 goat anti-rabbit and Alexa Fluor 488 goat anti-mouse (***iii***), or both primary and secondary antibodies (***iv***). DAPI dye (blue) in the mounting medium labels cell nuclei. The results reveal no fluorescent staining when either primary or secondary antibodies were excluded. Fluorescent staining was only detected when both antibodies were included in the incubation medium, indicating the specificity of the antibodies used in our study. The same experimental paradigm was conducted with Western blotting (***B***) procedures and protein bands were only detected when both the primary and secondary antibodies were used, further confirming the specificity of GPER antibody. Download Figure 7-1, TIF file.

### RNA sequencing data revealed different transcriptomic effects of GPER agonists on hippocampal and cortical neurons

**Figure 8.**
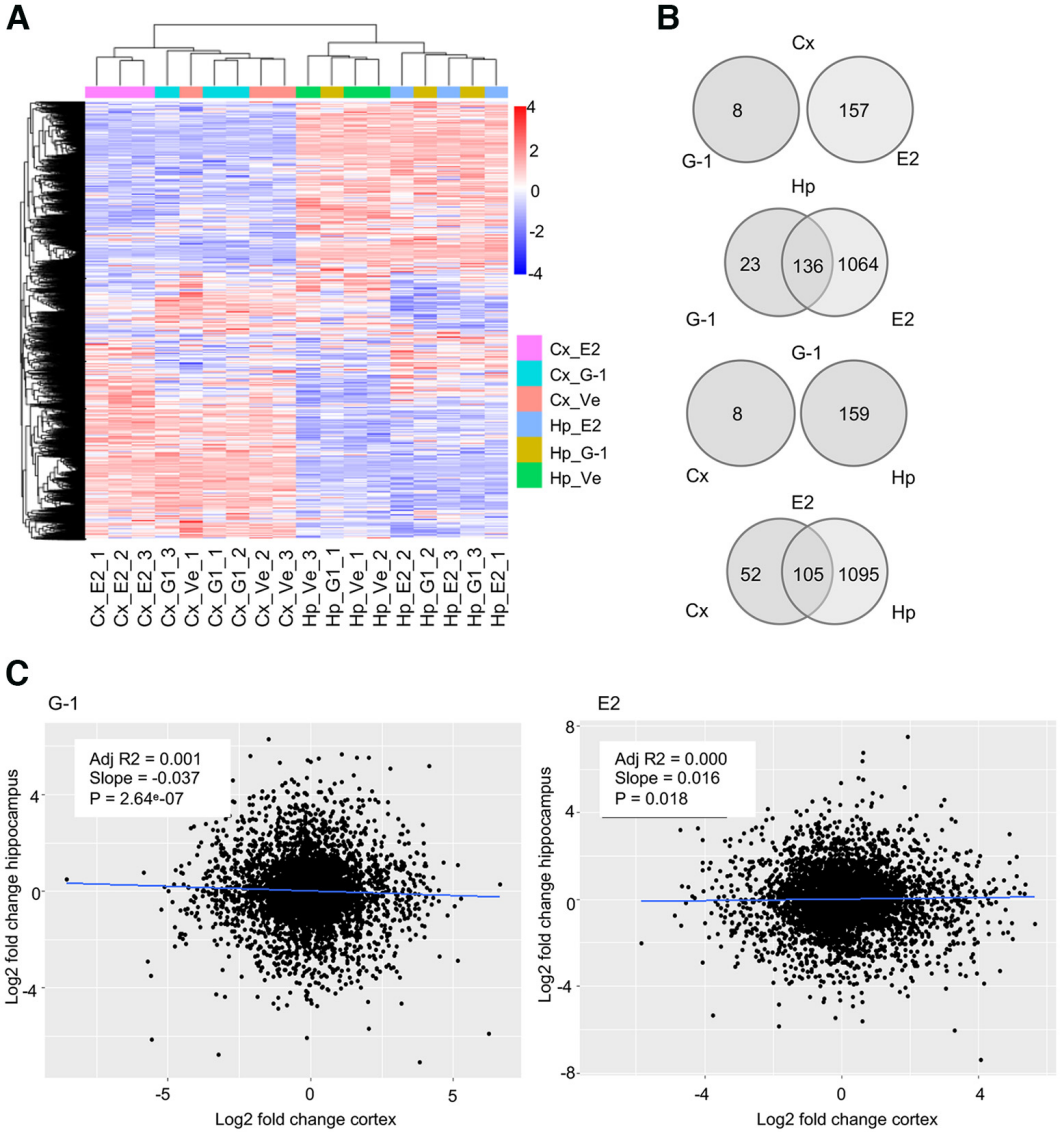
RNA sequencing data show different transcriptome changes in hippocampal versus cortical cultures after G-1 or E2 treatment. RNA sequencing was performed on hippocampal or cortical cultures treated with vehicle, G-1 (100 nm), or E2 (100 nm) for 72 h: a total of 18,749 genes were identified (*n *=* *3 for each group). ***A***, Heatmap showing the differential expression of genes in cortical (Cx) compared with hippocampal (Hp) cultures. The hierarchical clustering of the log2(fpkm + 1) values shows the separation of transcriptome between cortical and hippocampal cultures. Furthermore, the clustering reveals that in cortical cultures, G-1 treatment has little to no effect, clustering with vehicle, while in hippocampal cultures, both G-1 and E2 treatment are separated from vehicle. ***B***, Venn diagrams showing the overlap of significantly regulated genes among different primary cultures or treatments. In cortical samples, eight genes were significantly regulated by G-1 and 157 by E2 compared with the vehicle (*p *<* *0.005, FDR adjusted *p*-value) with no overlap between the treatments, indicating that in cortical cultures G-1 and E2 significantly regulate two distinct sets of genes. In hippocampal samples, 159 genes were significantly regulated by G-1 and 1200 by E2 compared with the vehicle (*p *<* *0.005, FDR adjusted *p*-value), and ∼85% (136 out of 159) of genes significantly regulated by G1 are also significantly regulated by E2 treatment. Moreover, there is no overlap between cortical and hippocampal cultures treated with G-1, while E2 significantly regulates 105 genes in both cultures. ***C***, Correlation plots show no correlation among cortical and hippocampal genes’ log2 fold change compared with vehicle after treatment with either G-1 or E2.

We conducted RNA sequencing experiments on G-1, E2, and vehicle-treated hippocampal and cortical cultures. Specifically, isolated rat E18 hippocampal and cortical neurons were cultured in vehicle (0.1% DMSO), G-1 (100 nm), or E2 (100 nm). After 72 HIC, RNA was extracted and sent to Novogene for RNA sequencing using the Illumina NovaSeq 6000 platform. For each group, three biological replicates were used and gene expression levels were normalized as fragments per kilobase of transcript sequence per million base pairs (FPKM). The observed transcriptomic changes were stronger in hippocampal compared with cortical cultures, but in both tissue cultures, E2 treatment yielded greater transcriptomic changes compared with G-1 treatment, indicating a broader, nonselective profile for E2. More specifically, in hippocampal cultures, the treatment of E2 yielded 1200 significantly differentially expressed (DE) genes compared with vehicle, while the specific GPER agonist G-1 had 159 significantly DE genes. In cultured cortical neurons, E2 led to 157 significantly DE genes and G-1 led to eight significantly DE genes. Interestingly, unsupervised hierarchical clustering using the log2(FPKM + 1) values showed a clear separation of transcriptomes between hippocampal and cortical cultures (Fig. 8*A*). The hierarchical clustering also revealed that G-1 and E2 treatment have a different outcome on the transcriptome in the two tissue cultures: in hippocampal primary cultures, both G-1 and E2-treated samples clustered separately from the vehicle control, while in cortical primary cultures, E2, but not G-1, treated samples clustered separately from the vehicle control. Moreover, Venn diagrams showed that in hippocampal cultures ∼85% of DE genes (136 out of 159 genes) were commonly regulated by G-1 or E2, while there was no overlap in the DE genes after G-1 or E2 treatment in cortical cultures (Fig. 8*B*). Next, we performed correlation plots, using the log2 fold change values, compared with vehicle controls, of E2 or G-1 in both cortical and hippocampal cultures (Fig. 8*C*). The log2 fold change values were used to correct for any difference in gene expression because of the different tissue cultures. The correlation plots further confirmed that both G-1 and E2 treatments yield very different transcriptome changes in the two different tissue cultures. Together, these data indicate that GPER action may affect the transcriptome profiles differently in hippocampal and cortical neurons, with a more robust effect on hippocampal than cortical neurons.

**Figure 9.**
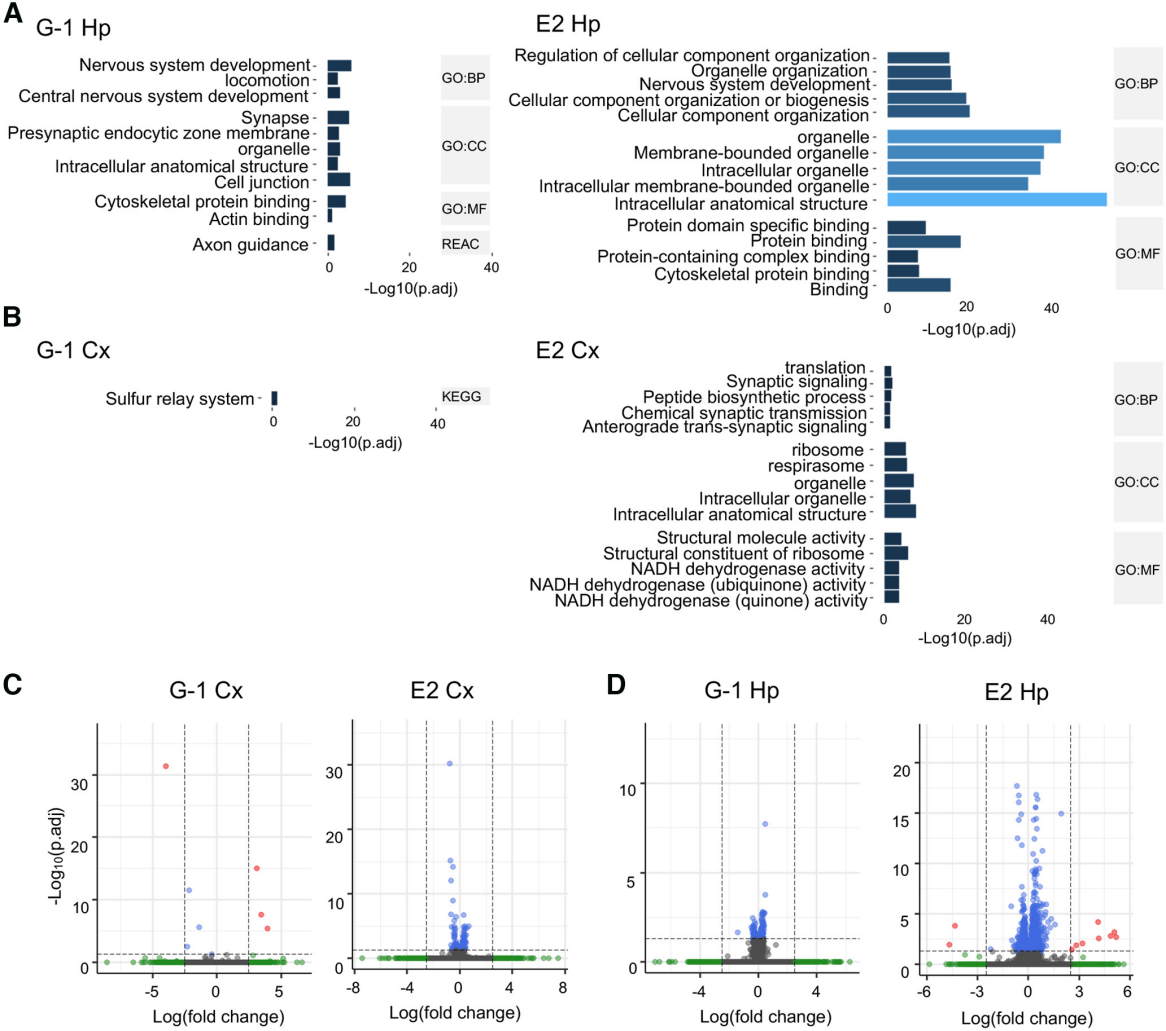
Pathway enrichment and correlation analysis show that GPER activation leads to specific changes in gene expression in hippocampal versus cortical neurons. ***A***, ***B***, Pathway enrichment analysis shows different enrichment of hippocampal (Hp) or cortical (Cx) samples after G-1 (100 nM) and E2 (100 nM) treatment. Only the top five significant enrichment terms ranked by *p*-value are shown (-Log10 adj. *p* > 1.3). ***A***, In hippocampal cultures, both G-1 and E2 are enriched for genes involved in nervous system development (GO:BP G-1, adj. *p* = 1.2e-06; E2, adj. *p* = 1.47e-16). Moreover, G-1 treatment shows enrichment for synapse (GO:CC, adj. *p* = 5.77e-06), cytoskeletal protein binding (GO:MF, adj. *p* = 4.01e-05) and axon guidance (KEGG, adj. *p* = 0.023). E2 treatment shows enrichment for organelle (GO:CC, adj. *p* = 2.32e-43) and protein binding (GO:MF, adj. *p* = 9.74e-19). ***B***, Transcriptome data from cortical cultures treated with G-1 show almost no enrichment, with only one significant GO term (KEGG: sulfur relay system). E2 treatment shows significant enrichment for synaptic signaling (GO:BP synaptic signaling, adj. *p* = 0.018; chemical synaptic transmission, adj. *p* = 0.047), ribosome (GO:CC, adj. *p* = 9.12e-06), and NADH dehydrogenase activity (GO:MF NADH dehydrogenase activity, adj. *p* = 0.0004). ***C***, ***D***, Volcano plots showing differentially expressed genes. The dashed line shows the *p *<* *0.05 cutoff (FDR adjusted *p*-value). Genes are represented as dots color-coded in gray (not significant, below log2 fold change threshold), green (above log2 fold change threshold > |2.5|), blue (significant, FRD adj. *p* < 0.05), and red (significant and above the log2 fold change threshold). The top DE genes in hippocampal and cortical cultures regulated by either G1 or E2 are provided in Extended Data Figures 9-1 (hippocampal) and 9-2 (cortical).

In addition, the Gene Ontology (GO) analysis, performed using gProfiler (Raudvere et al., 2019), revealed differences in enriched pathways in hippocampal and cortical cultures. Specifically, results from hippocampal cultures show that both G-1 and E2 significantly regulate pathways (indicated by asterisks) related to “nervous system development” (GO:BP G-1 adj. *p* = 1.2e-06, E2 adj. *p* = 1.47e-16) that includes the adhesion molecules genes *Nrcam* and *Adgrb2*, “intracellular anatomic structure” (GO:BP G-1 adj. *p* = 0.00, E2 adj. *p* = 1.55e-54) that includes the neurofilament protein gene *Nefm*, and “cytoskeletal protein binding” (GO:BP G-1 adj. *p* = 4.01e-05, E2 adj. *p* = 4.01e-05) that includes the actin-binding protein genes *Cfl1*, *Pfn1*, and *Fscn1*. In addition, E2 also showed significant enrichment for “organelle” (GO:CC adj. *p* = 2.32e-43) that includes synaptic vesicle genes such as *Syt4*, *Stxbp1*, and *Snap25* and “protein binding” (GO:MF adj. *p* = 9.74e-19) that includes the semaphoring genes *Sema3a*, *Sema5a*, and *Sema4c*. The G-1 group also showed significant enrichment for “axon guidance” (KEGG adj. *p* = 0.023) that includes genes involved in axon and dendrite growth like *Dpysl5*, *Dpysl3*, and *Gap43,* and “synapse” (GO:CC adj. *p* = 5.77e-06) that includes genes involved in signaling like *Calm2* and adhesion molecules like *Nrcam* (Fig. 9*A*, top five terms ranked by adjusted *p*-value). In cortical cultures, E2 treatment showed a significant enrichment for “synaptic signaling” (GO:BP synaptic signaling adj. *p*-value = 0.018; chemical synaptic transmission adj. *p*-value = 0.047) including genes like *Calm2* and *Syp*, “ribosome” (GO:CC adj. *p* = 9.12e-06) including ribosomal genes like *rps2*, *rps7*, *rps23*, *rpl9*, *rpl13*, and *rpl32*, and “NADH dehydrogenase activity” (GO:MF NADH dehydrogenase activity adj. *p* = 0.0004) including mitochondrial genes like *Mt-nd1*, *Mt-nd2*, *Mt-nd3*, *Mt-nd5*, and *Mt-nd6* (Fig. 9*B*, top five terms ranked by adjusted *p*-value). Interestingly, there is only one significant enrichment revealed for G-1 significantly regulated genes in cortical cultures (KEGG: sulfur relay system adj. *p* = 0.036) that includes only the cysteine desulfurase, mitochondrial-like gene LOC100911034. The slight effect of G-1 in cortical cultures is further shown by volcano plots (Fig. 9*C*). Lastly, our analysis of DE genes in hippocampal cultures also shows the significant regulation of GPCR signaling genes (*Rangap1* and *Gpsm1*) and protein kinase signaling genes (*Camk2b* and *Akt1)* in hippocampal but not cortical cultures. The list of the above-mentioned and other top DE genes in both cultures by either G1 or E2 was provided in Extended Data Figures 9-1 (hippocampal) and 9-2 (cortical).

10.1523/ENEURO.0475-21.2022.f9-1 Extended Data Figure 9-1 GO analysis of the differentially expressed genes in response to either G1 or E2 treatment in hippocampal cultures. Download Figure 9-1, XLS file.

10.1523/ENEURO.0475-21.2022.f9-2 Extended Data Figure 9-2 GO analysis of the differentially expressed genes in response to either G1 or E2 treatment in cortical cultures. Download Figure 9-2, XLS file.

## Discussion

This study sought to determine the role of GPER in the early neurodevelopment of rat E18 hippocampal and cortical neurons. Our experiments show that estrogen/GPER signaling exhibits strong neurotrophic effects through increased neurite growth in hippocampal, but not cortical, neurons. Activation of GPER also increases action potential firing and Ca^2+^ signaling more robust in hippocampal than in cortical neurons. RNA sequencing data further revealed significantly distinct DE genes and enriched pathways that are regulated by GPER activity in hippocampal versus cortical neuronal cultures. Specifically, this study identified potential molecular targets such as genes involved in axonal/dendritic growth, cytoskeletal binding, adhesion molecules, and G-protein/protein kinase signaling that may contribute to GPER’s neurotrophic effects in hippocampal neurons during early neuronal development. These results are important for our fundamental knowledge of estrogen functions via GPER signaling in different types of neurons during early nervous system development.

### Identification of GPER as a neurotrophic promotor for neurite outgrowth of rat E18 hippocampal, but not cortical, neurons

GPER has higher levels of expression in the brain relative to classical ERs (Hutson et al., 2019) and alteration of GPER or its signaling pathways has been found in patients with autism spectrum disorder and schizophrenia (Altun et al., 2017), suggesting that GPER may play a crucial role in the pathophysiology of these neuropsychiatric disorders. Research into autism and schizophrenia has looked at alterations at the neurodevelopmental level; however, there is limited research into understanding the neurodevelopmental impacts of targeting GPER. Here, we found that GPER activation increased neurite outgrowth, which is consistent with previous studies showing that E2 increased neuritogenesis in primary mouse E17 hippocampal neurons via GPER activation (Ruiz-Palmero et al., 2011, 2013). Earlier studies have demonstrated that E2 exposure increases the number and length of neuritic filopodia in primary rat E18 hippocampal neurons within minutes of exposure, although it is not clear whether this action is mediated by GPER because of the lack of knowledge of this receptor at that time (Brinton, 1993). Nevertheless, these studies indicate a neurotrophic role of GPER in promoting early development in hippocampal neurons.

In cortical neurons, our and other studies have shown that the effect of estrogen or GPER activation/inactivation appears more variable in magnitude and direction in terms of neurite outgrowth. Brinton et al. (1997) demonstrated that E2 differently regulates cortical neural outgrowth with a significant enhancement in parietal and occipital neurons, no significance in frontal neurons, and an inhibitory effect on temporal neurons. Furthermore, these results were not inhibited by the nuclear ER antagonist ICI 182780, suggesting that there may be a GPER-related mechanism. Consistently, our rat E18 mixed cortical neurons revealed highly variable effects in terms of the magnitude (potency) and direction (stimulation vs inhibition) of GPER agonists such as E2 and G-1.

Interestingly, our results also showed the GPER antagonist G-15 alone significantly inhibited cortical, but not hippocampal, basal outgrowth. It is not known whether the inhibitory effect of G-15 is because of nonspecific binding to nuclear ERs, which are important for maintaining the basal growth of cortical neurons. However, the 10 nm G-15 we used is far below the concentration (10 μm) for nonspecific binding to ERs (Dennis et al., 2011). This then poses other possibilities that may explain the observed differences in hippocampal and cortical neurons in response to G-15. For example, GPER displays greater constitutive activity in cortical neurons than in hippocampal neurons, which may be related to either an increase in expression of the transducer associated with receptor signaling or through a greater degree of endogenous synthesis of estrogen by cortical neurons (Cornil et al., 2006; Hojo et al., 2008; Azcoitia et al., 2011). Receptors with high levels of constitutive activity may experience a reduction in basal activity (i.e., inverse agonist) in response to antagonist (Kenakin, 2006). This may explain the observed inhibitory effects of G-15. Together, variable expression of transducers and endogenous synthesis of GPER ligands may explain the differential response of GPER to G-15 in hippocampal and cortical neurons. The impact of these observations may result in distinct physiological outcomes and future experimental testing of these postulations is warranted.

### Different actions of GPER on neuronal activity and cytosolic Ca^2+^ signaling in hippocampal and cortical neurons

The rapid action and signaling of GPER in neurons have been associated with the ability to change neuronal firing activity and/or intracellular messengers such as Ca^2+^ (Altmann et al., 2015; Evans et al., 2016; Rebas et al., 2017). The frequency and patterns of neural activity and Ca^2+^ oscillations are important signaling factors that influence early neuronal development, including growth cone dynamics, axon pathfinding, neurite extension, and synaptic plasticity (Kanemaru et al., 2007; Toth et al., 2016). Here, we found that GPER induced a rapid increase in neuronal firing activity within minutes of E2 and G-1 application in both hippocampal and cortical neurons. Interestingly, GPER significantly increased intracellular Ca^2+^ in hippocampal, but not cortical, neurons. Consistently, E2 has been found to increase cytosolic Ca^2+^ in hippocampal neurons (Wu et al., 2005), while a recent study failed to detect an effect of GPER on cortical Ca^2+^ in neurons, but discovered an effect in astrocytes (Roque and Baltazar, 2019; Roque et al., 2019). GPER also increases cytosolic Ca^2+^ in hypothalamic astrocytes (Kuo et al., 2010). Together, these studies indicate that GPER’s role in regulating neuronal excitability and intracellular Ca^2+^ is brain region and cell type dependent.

In addition, the source of Ca^2+^ and Ca^2+^ signaling pathways may be unique to brain regions or cell types. We found that G-1 activity increases cytosolic Ca^2+^ likely via VGCC-mediated extracellular Ca^2+^ entry and PLC-dependent internal Ca^2+^ store release. This is consistent with the finding that G-1 induced cytosolic Ca^2+^ in brain microvascular endothelial cells via L-type VGCCs (Altmann et al., 2015). Interestingly, the increase in cytosolic Ca^2+^ in endothelial cells further activated Ca^2+^-activated K^+^ channels, contributing to G-1-induced hyperpolarization (Altmann et al., 2015). This is in contrast to the membrane depolarization observed in our study and another with SH-SY5Y cells (Ding et al., 2019). It is important to note that CdCl_2_ and Ca^2+^-free solutions used in this study are nonselective blockers and may affect other channel activities, such as K^+^ channels (Stockand et al., 1993; Du et al., 2011) and SOCs. Therefore, the involvement of specific VGCCs and other potential channels such as K^+^, SOCs, etc. should be further explored using selective blockers and molecular knock-out methods. Nevertheless, the degree of change in neuronal firing activity and/or Ca^2+^ has been tightly associated with developmental processes such as synaptic plasticity (Malenka, 1991; Zamora Chimal and De Schutter, 2018) and axon guidance (Sutherland et al., 2014). Thus, the different GPER actions and potency on neural activity and Ca^2+^ may contribute to the difference in neurite outgrowth in hippocampal and cortical neurons. Our data indicated that Ca^2+^ release via PLC-IP_3_ stores may participate in maintaining Ca^2+^ spiking patterns because the amplitude and spiking pattern of Ca^2+^ (Fig. 6*E*) were drastically reduced in the presence of selective PLC inhibitors. The identification of major signaling pathways associated with GPER allows us to better understand its physiological roles and mechanistic actions during early neuronal development.

### GPER expression in hippocampal and cortical neurons or tissues

To test whether GPER expression in hippocampal and cortical neurons may contribute to their distinct physiological effects, we found that GPER is profoundly expressed in both cell types, more richly expressed in the cell body than in neurites, and primarily localized intracellularly. Although we did not find a difference in GPER expression pattern between hippocampal and cortical neurons, our results revealed prevalent subcellular localization of GPER. This agrees with previous results in adult hippocampal (Matsuda et al., 2008) and hypothalamic (Sakamoto et al., 2007) neurons, as well as in COS7 (Monkey kidney fibroblast) cells (Revankar et al., 2005), although plasma membrane localization was found in intact (fixed but not permeabilized) HEK-293 expressing HA-GPER (Filardo et al., 2007). Interestingly, the same study also showed that when HEK cells were permeabilized with Triton X-100, intracellular HA-GPER clusters were found. It is not known whether the observed intracellular localization of GPER in our study is a consequence of permeabilization during immunostaining. However, intracellular localization of GPER and many other GPCRs has been reported and implicated to be associated with receptor biogenesis, posttranslational regulation, and trafficking (Bermak and Zhou, 2001; Mizrachi and Segaloff, 2004; Duvernay et al., 2005; Revankar et al., 2005). Many GPCRs including GPER are intracellularly functional, particularly when their ligands (e.g., steroid hormones) are membrane permeable (Nezhady et al., 2020). Subcellularly, GPER was shown to be localized in cytosolic organelles such as Golgi, endoplasmic reticulum, and other endosome or lysosomal organelles (Gaudet et al., 2015). Intriguingly, previous studies have reported that >30 different GPCRs including GPER were also detected in the nuclei of cells (Gobeil et al., 2006; Boivin et al., 2008; Pupo et al., 2013, 2017; Joyal et al., 2015), where it was shown to be localized to the nucleus of isolated breast cancer-associated fibroblasts (CAFs) and functions as a transcription factor that up-regulates the expression of genes such as *c-FOS* or *CTGF* (connective tissue growth factor; Madeo and Maggiolini, 2010; Pupo et al., 2013). Consistently, GPER in our cultured E18 hippocampal and cortical neurons also had nuclear localization (Fig. 7), and other studies found that the GPER protein sequence contains a nuclear localization signal and de-glycosylation of GPER triggers nuclear localization (Pupo et al., 2017). The existence of nonglycosylated (∼42 kDa) and glycosylated (>42 kDa) GPER proteins was found to correlate with various protein species in immunoblotting studies (de Valdivia et al., 2019). Similarly, in E18 hippocampal and cortical tissues, we observed two protein species with mass sizes of ∼42 and 50 kDa. Since our GPER antibody recognizes both protein species, it is not known whether these different protein species are selectively localized in the cytoplasm versus nuclear area in our fluorescence images. Future studies revealing the specific localization of two GPER protein forms in the subcellular structures and their associated functions would be interesting. Regardless of their localization patterns, our qPCR and Western blot data show predominant expression of GPER transcripts and proteins in the cortex compared with the hippocampus. This result is consistent with a previous study showing that GPER mRNA level is more abundant in cortical than hippocampal tissues of both female and male adult rat brains (Hutson et al., 2019). These expression data seem to indicate that the amount of GPER expression does not correlate with the robustness of physiological response it may induce. The physiological effects we observed may instead be determined by the coupling of GPER to different signaling pathways and molecular effectors in hippocampal and cortical neurons.

### Distinct transcriptomic regulation by GPER in hippocampal and cortical neurons

Correlating with the distinct outgrowth and Ca^2+^ signaling effects, our RNA sequencing revealed distinct transcriptomic regulation by GPER in hippocampal and cortical neurons, resulting in different gene clustering patterns between treatments in hippocampal and cortical cultures. As shown by unsupervised hierarchical clustering analysis, there was no significant correlation between significantly DE genes as shown by correlation plot analysis and little overlap as shown by Venn diagram analysis. These data indicate that few shared genes and pathways are commonly upregulated or downregulated in both hippocampal and cortical neurons by either E2 or G-1. Importantly, our data clearly demonstrate that E2 and G-1 induced a much stronger transcriptomic regulation of DE genes in hippocampal (1200/159 DE genes) than cortical (157/8 DE genes) cultures, indicating that estrogen and ERs, especially GPER, seem to be more actively engaged in transcriptomic regulation in hippocampal cells at the E18 stage. More interestingly, our hippocampal RNAseq data show transcriptomic enrichment for genes/pathways critically involved in early brain developmental processes such as axonal/dendritic growth and G-protein/protein kinases signaling. The enrichment of DE genes in these early developmental processes supports the neurotrophic effects of G-1/E2 on promoting hippocampal neuritogenesis, neurite outgrowth, and filopodia extensions in our and other studies (Brinton, 1993; Ruiz-Palmero et al., 2011, 2013). Although G-1 and E2 did not promote neuritic development of E18 cortical neurons, our RNA sequencing data indicate a role of E2 in regulating transcripts related to protein translation, chemical synaptic signaling, and ribosomal and mitochondrial pathways. In addition, G-1 treatment was found to significantly regulate a cysteine desulfurase, mitochondrial-like gene, LOC100911034, which catalyzes the formation of an iron-sulfur (Fe-S) cluster (Poliak et al., 2010) that acts as an essential protein cofactor for many crucial biochemical processes (Patra and Barondeau, 2019). Together, these findings in cortical neurons indicate that, although GPER did not significantly affect early neurite outgrowth, it may play essential roles in regulating biochemical processes or later synaptic events such as chemical synaptic signaling and protein synthesis during synaptic plasticity. In support of this hypothesis, studies have shown that aromatase, a member of the cytochrome P450 superfamily that is responsible for estrogen biosynthesis, is expressed and colocalized with both presynaptic and postsynaptic machinery in cultured mature cortical neurons (Srivastava et al., 2010). This study, together with our RNA sequencing results, suggests an important role for estrogen in later developmental stages, spinogenesis, and plasticity in cortical neurons.

In hippocampal cultures, many DE genes that are significantly regulated by G-1 and/or E2 are related to nervous system development and particularly worthy of discussing here, including genes involved in neurite growth such as *Dpysl5*, *Dpysl3*, and *Gap43*. The *Dpysl5/3* (*dihlikeydropyrimidinase-related protein 5/3*) gene encodes a member of the collapsin response mediator protein (CRMP) family that is highly expressed in the olfactory bulb and hippocampus of developing brains and is involved in axon guidance and neurite outgrowth during neural development (Veyrac et al., 2011; Gong et al., 2016). CRMP members are regulators of voltage-gated Ca^2+^ and Na^+^ channels (Chew and Khanna, 2018) and hence neuronal activity and activity-dependent neurite outgrowth (Wilson et al., 2014). *Gap43* encodes Gap43, which is a critical component of the axonal growth cone and presynaptic terminal (Strittmatter et al., 1994; Okada et al., 2021). Interestingly, the expression of *Gap43* mRNA in neurons can be modulated by neuronal activity (Caprini et al., 2003; Rosskothen-Kuhl and Illing, 2014). The ability of CRMP and GAP43 to regulate neurite development and activity is intriguing since GPER agonists are found to induce activity changes in hippocampal neurons by the present study. In addition, several genes encoding actin-binding proteins (*Cfl1*, *Pfn1*, *Fscn1*), neurofilament (*Nefm*), and adhesion molecules (*Nrxn2*, *Adgrb2*, and *Cadm4*) are also significantly upregulated by G-1 and E2 or G-1 alone (also see Extended Data Figs. 9-1, 9-2; Hunter et al., 2011; Golan et al., 2013; Duman et al., 2016; Pervolaraki et al., 2019). In addition, the expression of *Cfl1*, *Pfn1*, and *Nrxn2* genes are found to be activity or Ca^2+^ dependent (Neuhoff et al., 2005; Rozic-Kotliroff and Zisapel, 2007; Feuge et al., 2019; Liakath-Ali and Südhof, 2021) and estrogen has been discovered to stimulate the phosphorylation of Cofilin (encoded by *Cfl1* gene), which induces elongation of actin filaments and growth of dendritic spines (Yuen et al., 2011). Furthermore, our RNAseq results also point to GPCR signaling genes specifically altered in hippocampal cultures. For example, both G-1 and E2 treatments increase the expression of *Rangap1* (Ran GTPase activating protein 1) which is involved in Ras signaling (Takeda et al., 2005; Ritterhoff et al., 2016), and of *Gpsm1* (G-protein signaling modulator 1) which is involved in the regulation of Gα_i/o_ or Gβγ subunits through GPSM1 binding to GDP-bound Gα leading to the release of free Gβγ (Blumer et al., 2012; Oner et al., 2013). Gβγ has been shown to directly inhibit VGCC (Tedford and Zamponi, 2006) or indirectly release Ca^2+^ from internal stores via PLC activation (Boyer et al., 1992; Park et al., 1993; Werry et al., 2003). These effects of Gβγ on cytosol Ca^2+^ may mediate different and opposing effects in different cell types such as in cortical neurons versus glial cells (Roque and Baltazar, 2019; Roque et al., 2019) or hippocampal neurons seen in this study. Future studies on GPER’s association with G_s_ or G_i/o_ as well as their Gβγ signaling in hippocampal and cortical development and physiology would be interesting and important. Lastly, two protein kinase genes, *Camk2b* and *Akt1*, are significantly upregulated by G-1 or E2. Camk2b encodes Ca^2+^/calmodulin-dependent protein kinase II β (CaMKIIB), which is involved in the regulation of neurite extension and dendritic arborization in developing hippocampal neurons (Wayman et al., 2006; Puram et al., 2011). Since Ca^2+^ is a major determinant for GPER-distinct effects on hippocampal and cortical neurons, a downstream Ca^2+^ effector such as CaMKIIB appears to be an intriguing kinase for future follow-up studies. Furthermore, G-1 (but not E2) treatment significantly increases the expression of *Akt1*, which encodes protein kinase B (PKB) that is involved in multiple functions in neurons via activation of the nuclear factor κB (NF-κB) transcription factor or mammalian target of rapamycin (mTOR) pathways (Bai et al., 2009; Manning and Toker, 2017).

In conclusion, this study identified that GPER plays a more prominent neurotrophic role in the neurite outgrowth of rat E18 hippocampal than cortical neurons. This may arise from the signaling events of GPER that have greater effects on the rapid modulation of neuronal activity and Ca^2+^ oscillations. The resulting change in activity and Ca^2+^ then act as determining signals for downstream gene transcriptomic regulation and morphologic development. Our data also indicates that GPER may regulate neurite outgrowth in hippocampal neurons by actively regulating gene profiles involved in nervous system development, axonal/dendritic growth, and G-protein/kinase signaling. This study identifies several interesting genes and signaling pathways that warrant further research. While our data agreed with previous studies showing a lack of activity or variable effect in response to GPER activation within the cortex, these results do not rule out the important role of GPER in cortical neurons. Rather, we emphasize that the involvement of GPER may depend on developmental period, cell type, or brain region. Identifying these differences and response pathways is crucial for our understanding of estrogen function via GPER in early neuron development or neurodevelopmental disorders.

## Synthesis

Reviewing Editor: Matthew Grubb, King’s College London Decisions are customarily a result of the Reviewing Editor and the peer reviewers coming together and discussing their recommendations until a consensus is reached. When revisions are invited, a fact-based synthesis statement explaining their decision and outlining what is needed to prepare a revision will be listed below. The following reviewer(s) agreed to reveal their identity: Van Lu.

Reviewer 1:

Strengths:

This is a well-rounded study examining multiple aspects of hippocampal and cortical neuron development and activity and the effects of membrane-estrogen receptors, GPER, in neuronal developement. Both G-1, a selective GPER agonist, and estrogen, E2 non-selective agonist of GPER and nuclear estrogen receptors, were tested. The inclusion of the inhibitor G-15 is a strength, but a control to assess the selectivity to GPER would alleviate anecdotal concerns over this inhibitor’s selectivity. Overall, the study’s results support their conclusions.

I have some recommendations for the authors to improve the scientific rigor of their study.

- Bar graphs do not show individual data points. To help assess the variability in system should represent individual data points on top of bar graphs. Also, how many times experiments repeated? N indicates total number of neurons but across how many preparations to account for any litter to litter variability.

- Analysis: Measuring neurite outgrowth. How were neurons selected, randomly selected neurons? To avoid bias, analyzer should be blinded to which treatment group was analyzed or if using an automated program state so. The same with MEA recordings, analyzed blinded to groups?

- Missing controls: Not all studies examined the effect of G15 inhibitor alone such as measurement of neurite outgrowths. Also, GPER-mediated Ca2+ responses no Cd2+ or U73122 alone groups.

- Quantifying GPER expression using a more quantitative measurement (RT-qPCR, WB). Immunofluorescence has its challenges in quantifying protein levels (e.g. non-linear amplification of signal by PMT). Plus, the staining of GPER is membrane bound in cortex vs cytoplasmic in hippocampal neurons. As a membrane localized receptor, it would be expected to be concentrated at the membrane which would likely give a lower signal ratio to nuclear DAPI stain. The localization of GPER in the cytoplasm in hippocampal neurons would need to be explained too. Also, the exemplar images of cortical neurons stained with beta-tubulin is very different from the images in Fig1 illustrating measurements of neurite outgrowth. Again, adding in the individual data points would help illustrate the variability across neurons or else the authors would have to explain why such differences were found.

Reviewer 2:

This manuscript studied the effects of selective (G-1) and non-selective (E2) agonists of the G-protein coupled estrogen receptor, either alone, or combined with receptor antagonists, on their ability to stimulate outgrowth, firing, calcium oscillations, and differential gene expression in embryonic hippocampal and cortical neurons. This manuscript is generally well written and the figures are laid out clearly.

Major points:

1. The selectivity of the agonists (eg. G-1) and antagonists (G-15) (as well as other pharmacological tools such as DDA, U73343 etc) should be discussed and evidence for their selectivity at the concentrations used, referenced in the Introduction or Methods section (especially for the GPER agonists/antagonists). Proper interpretation of the findings eg. effects on neurite outgrowth depends on knowing how selective the GPER agonists / antagonists are for GPER, particularly at the concentrations used in this study.

Why did use of G-15 inhibit neurite outgrowth in cortical cells (only)? The possibilities for this should be discussed more thoroughly. The authors suggested there may be some other endogenous factors binding to GPER. However, knowing whether G-15 is a competitive or non-competitive antagonist could shed more light on whether this is likely. The authors should also consider other explanations, such as whether G-15 may have non-selective actions, or whether there is any evidence that GPER has constitutive actions. Is it known to interact with other G-protein coupled receptors? The n’s for the neurite outgrowth experiments should also be included in the text.

Also, why did G-15 not completely block the effects of G-1 on firing, but only in cortical cells? The possibilities for this finding were also not fully discussed.

Both of these issues should be addressed in more detail in the discussion and possible explanations for the results provided.

2. As the effects of the agonists / antagonists on firing activity were expressed as “normalized” number of spikes, it would be useful information to know whether there was any difference in the baseline level of spiking (before the treatments) between the cell types. These data could be provided, as if there were any differences in baseline activity, this could potentially affect the outcome of the treatments. It also wasn’t clear what the n values represented in this experiment. Eg. Line 293 “n = 60 for all conditions”. Is this a total n = 60 for all conditions combined, or for each condition. Also, does the n value represent total number of cells? How many brains were used to generate the cells for each treatment and how many trials were conducted? How many cells from different brains were tested in each condition (repeats) is important information that should be included in the methods / results. If all cells tested in one condition were only from one brain and recorded with only one trial (in one dish), this could be problematic.

3. There are a number of places in the manuscript where ANOVA results are not provided (eg. effects of DDA and U73343 on calcium levels) and only post-hoc data appear to be given. ANOVA results should be provided for all data sets (in the results or figure legends), and degrees of freedom (df)should also be included with all ANOVA results throughout the manuscript. Dfs also provide important information, without which, it is harder to judge the statistical results.

4. There do not appear to have been any control experiments performed for the antibody staining experiments. How specific are these antibodies? Were any controls performed, (e.g exclusion of primary antibody, or better yet, a pre-adsorbed control)? There needs to be some evidence provided for the specificity of these antibodies for GPER - either referenced from other studies where this has clearly been shown in rat embryonic neurons - or reported in this study, as the quantification and hence the interpretation using this method depends on this. Furthermore, it is not clear to me why the authors used a normalization of the GPER signal to DAPI? Any inter-experiment variability in antibody staining for GPER is unlikely going to be resolved using a DNA stain. What is the rationale and justification for this approach? If the imaging parameters are kept consistent between experiments, why would normalization to DAPI be required? This needs to be justified clearly in the methods / results.

Did the authors consider taking brain samples from hippocampus and cortex and running Western Blots (where protein loading can be controlled) to quantify the differences in GPER expression between the two brain regions?

If appropriate specificity of the antibody cannot be fully demonstrated from either these studies (with appropriate controls added) or previous studies on rat embryonic neurons, then the authors should tone down their claims of quantitative differences in receptor expression.

Minor changes:

1.The introduction laid out the background and rationale to the study nicely. My only suggestion would be to provide more information about what species were used in many of the studies described. It often wasn’t clear (eg. in section from lines 64 to 86) when discussing background literature on GPER and agonists, whether the research had been carried out in rodents or in other species.

2.Line 257-258, states no difference between any groups “compared to control or vehicle”. I only see a vehicle control group in the data and figures. Is there another control group used (perhaps without vehicle)? Line 246-247 stated either vehicle or agonists/antagonists were added to culture, but does not mention any other type of control.

3.The subheadings (276, 306) should more accurately state “GPER agonists”, because as written, the authors are assuming that the GPER activity is solely responsible for the effects of E2 or G-15.

4. There were a number of typos / grammatical errors throughout the manuscript that need to be corrected. The definite /indefinite article (the/a) is also required in a number of places. A presumable “auto-correct” of “mediated” to “medicated” needs to be corrected in a number of places too.

5. G-protein βγ subunits are also known to directly inhibit VGCCs, and this effect should be mentioned too, as it is quite possible that the receptor could be mediating different, even opposing effects, in different cell types.

6. Line 356: The data from figure 6 are misrepresented in text (eg. states Fig 6A-D, where data on PLC inhibition are actually shown in Fig 6E-F). I actually recommend switching the order of the figures such that the PLC data is shown before Ca-free saline and cadmium, to align with the order presented in the text. The statistical reporting in Fig 6 (graph) is quite messy and inconsistent. Could * located directly above a data bar represent a difference to vehicle control in all graphs in this manuscript (as this has been used at times, but not at others, and so there is inconsistency in stats reporting in the figures). Then other differences between groups could be represented with the lines?

7. The authors make some claims that are not fully supported by their data and should likely be toned down (eg. line 562, “RNA sequencing data indicate a “critical” role of E2; line 568: GPER may play “essential” roles).

8. Line 570: the implications of aromatase mentioned in this statement and how it links to the previous sentences should be made clearer.
