# Supplementary figures and images for: Differential Effects of the G-Protein-Coupled Estrogen Receptor (GPER) on Rat Embryonic (E18) Hippocampal and Cortical Neurons
Source: eNeuro. 2022 Jul 14;9(4):ENEURO.0475-21.2022. doi: 10.1523/ENEURO.0475-21.2022 (PMC9291730; doi:10.1523/ENEURO.0475-21.2022)

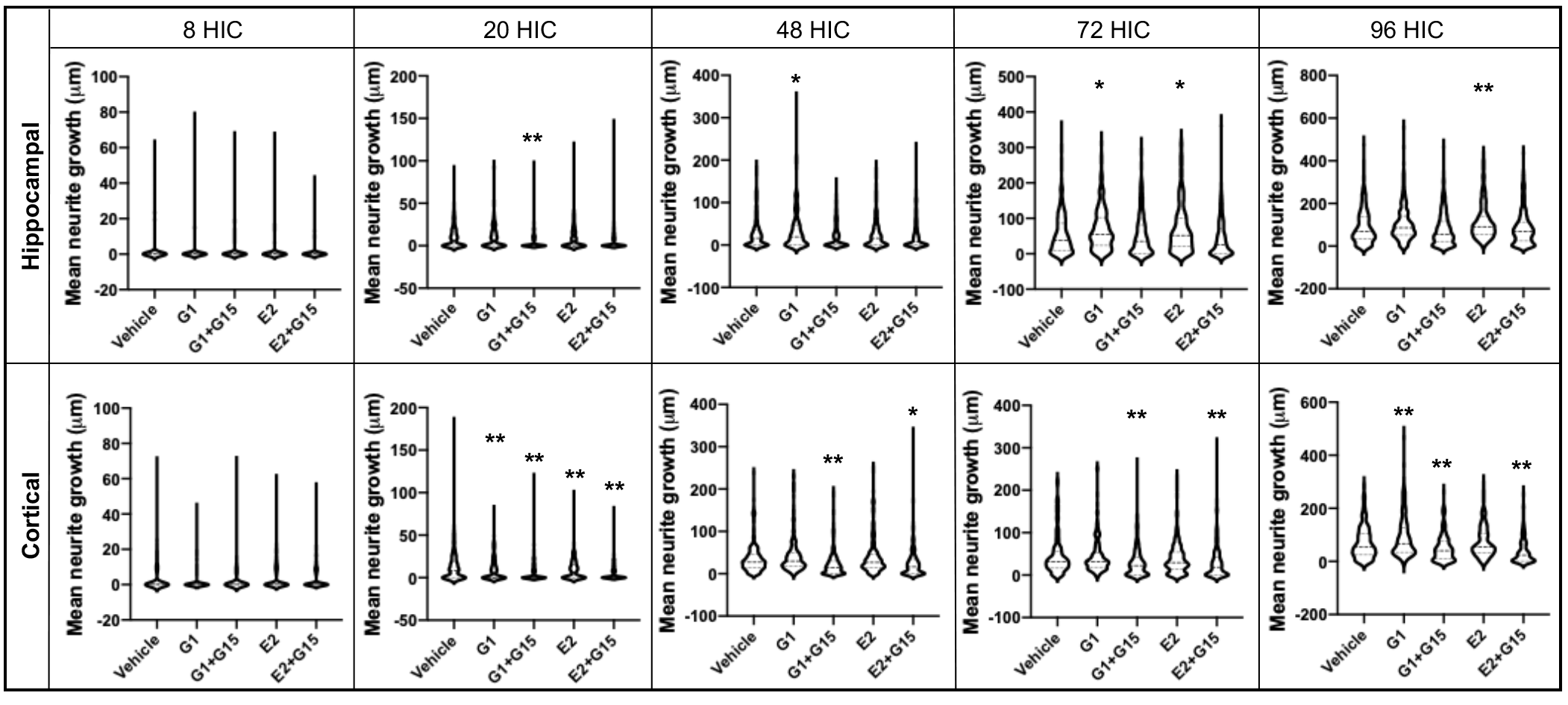

Supplement: Extended Data Figure 1-1 — GPER agonists increase neurite outgrowth in hippocampal but not cortical neurons. GPER activation by a selective agonist, G-1, and nonselective agonist, E2, increases neurite outgrowth in hippocampal neurons at multiple time points as specified as HIC, and blocking GPER activation with a selective antagonist, G-15, inhibits this effect. In cortical neurons activation of GPER does not appear to enhance outgrowth except at 96 HIC, but blocking GPER activation inhibited neurite outgrowth throughout; *p < 0.05 versus vehicle, **p < 0.01 versus vehicle. Download Figure 1-1, TIF file. [file enu-eN-NWR-0475-21-s02.tif]

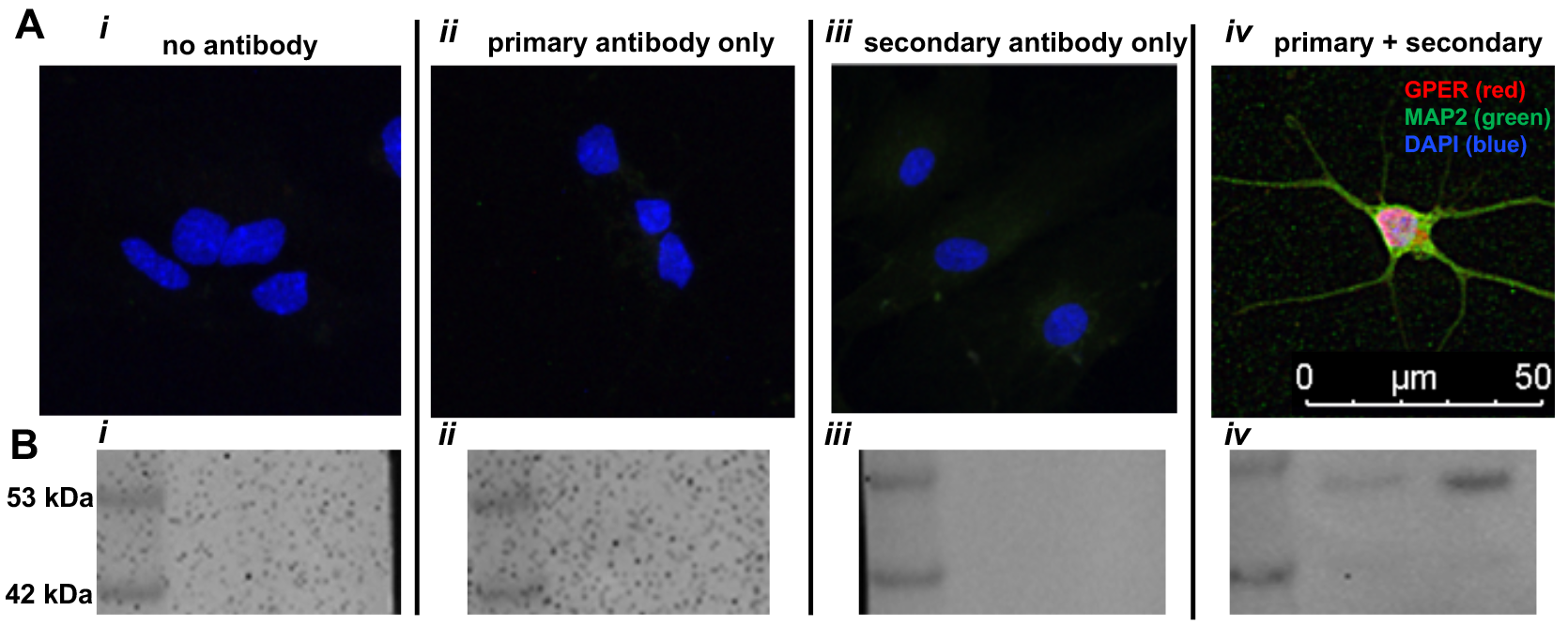

Supplement: Extended Data Figure 7-1 — Validation of antibody specificity by immunofluorescent and Western blot experiments. A, To verify the specificity of GPER (red) and MAP2 (green, neuronal marker) antibodies used in the fluorescence studies, neurons were fixed and immunostained in incubation medium with no antibody (i), only primary antibodies for GPER and MAP2 (ii), only secondary antibodies for Alexa Fluor 568 goat anti-rabbit and Alexa Fluor 488 goat anti-mouse (iii), or both primary and secondary antibodies (iv). DAPI dye (blue) in the mounting medium labels cell nuclei. The results reveal no fluorescent staining when either primary or secondary antibodies were excluded. Fluorescent staining was only detected when both antibodies were included in the incubation medium, indicating the specificity of the antibodies used in our study. The same experimental paradigm was conducted with Western blotting (B) procedures and protein bands were only detected when both the primary and secondary antibodies were used, further confirming the specificity of GPER antibody. Download Figure 7-1, TIF file. [file enu-eN-NWR-0475-21-s03.tif]
